# Semaphorin 3F signaling actively retains neutrophils at sites of inflammation

**DOI:** 10.1172/JCI130834

**Published:** 2020-05-18

**Authors:** Tracie Plant, Suttida Eamsamarng, Manuel A. Sanchez-Garcia, Leila Reyes, Stephen A. Renshaw, Patricia Coelho, Ananda S. Mirchandani, Jessie-May Morgan, Felix E. Ellett, Tyler Morrison, Duncan Humphries, Emily R. Watts, Fiona Murphy, Ximena L. Raffo-Iraolagoitia, Ailiang Zhang, Jenna L. Cash, Catherine Loynes, Philip M. Elks, Freek Van Eeden, Leo M. Carlin, Andrew J.W. Furley, Moira K.B. Whyte, Sarah R. Walmsley

**Affiliations:** 1 University of Edinburgh Centre for Inflammation Research, Queen’s Medical Research Institute, University of Edinburgh, Edinburgh, United Kingdom.; 2 Department of Infection, Immunity and Cardiovascular Disease and; 3 Bateson Centre, University of Sheffield, Sheffield, United Kingdom.; 4 BioMEMS Resource Centre, Division of Surgery, Innovation and Bioengineering, Department of Surgery, Massachusetts General Hospital, Harvard Medical School, Boston, Massachusetts, USA.; 5 Burn Care, Shriners Hospitals for Children — Boston, Boston, Massachusetts, USA.; 6 Cancer Research UK Beatson Institute, Glasgow, United Kingdom.; 7 Department of Biomedical Science, University of Sheffield, Sheffield, United Kingdom.; 8 Institute of Cancer Sciences, University of Glasgow, Glasgow, United Kingdom.

**Keywords:** Inflammation, Pulmonology, Cell migration/adhesion, Cellular immune response, Neutrophils

## Abstract

Neutrophilic inflammation is central to disease pathogenesis, for example, in chronic obstructive pulmonary disease, yet the mechanisms that retain neutrophils within tissues remain poorly understood. With emerging evidence that axon guidance factors can regulate myeloid recruitment and that neutrophils can regulate expression of a class 3 semaphorin, SEMA3F, we investigated the role of SEMA3F in inflammatory cell retention within inflamed tissues. We observed that neutrophils upregulate SEMA3F in response to proinflammatory mediators and following neutrophil recruitment to the inflamed lung. In both zebrafish tail injury and murine acute lung injury models of neutrophilic inflammation, overexpression of SEMA3F delayed inflammation resolution with slower neutrophil migratory speeds and retention of neutrophils within the tissues. Conversely, constitutive loss of *sema3f* accelerated egress of neutrophils from the tail injury site in fish, whereas neutrophil-specific deletion of *Sema3f* in mice resulted in more rapid neutrophil transit through the airways, and significantly reduced time to resolution of the neutrophilic response. Study of filamentous-actin (F-actin) subsequently showed that SEMA3F-mediated retention is associated with F-actin disassembly. In conclusion, SEMA3F signaling actively regulates neutrophil retention within the injured tissues with consequences for neutrophil clearance and inflammation resolution.

## Introduction

Effective host responses to injury and infection require both rapid recruitment of neutrophils into tissues and timely inflammation resolution. Research efforts have focused separately on the initiation and resolution phases of inflammation and, in terms of resolution, on how neutrophil survival responses determine the duration and extent of the inflammatory response (1). Less focus has been placed on mechanisms by which viable neutrophils may be retained within the inflammatory site and thus contribute to the ongoing inflammation, yet this may be of particular therapeutic importance given the dominance of viable neutrophil numbers in inflamed tissue, even during the resolution phase of the innate immune response.

Directed neutrophil migration, essential for neutrophil recruitment to the injury site and an effective innate immune response, is critically dependent upon polarization of the cell. Cell polarity is tightly regulated by phosphoinositide 3-kinase (PI3K) signaling products, with PI3K activation at the leading edge linked to microtubule assembly and activation of key regulators of the actin cytoskeleton, including Rho and Rac (2–4). These pathways are, unsurprisingly, well conserved across cell populations in which directed migration of cells is also of critical importance, for instance, axonal migration or angiogenesis (5–7). Less is known about the mechanisms that regulate neutrophil speed of migration to, and maintenance at, the site of tissue injury. Given the parallels to directed migration in other systems, we asked whether proteins previously shown to regulate either axon repulsion or attraction and displaying regulated expression in immune cells may be important in signaling neutrophil retention within inflamed tissues and thus have an impact on inflammation resolution.

In previous studies of neutrophil hypoxic responses (8), we observed that one of the genes most highly regulated by the prolyl hydroxylase domain–containing enzyme PHD3 was *Sema3f*, a member of the semaphorin family of secreted and transmembrane axon guidance molecules (9). Semaphorins, originally identified as chemorepulsive molecules for axonal growth cones, have since been implicated in regulating cell motility in the context of vascular growth and tumor progression, and in immune signaling and immune synapse formation (10–12). More recently, the class 3 semaphorin SEMA3A was shown to act as an attractant for tumor-associated macrophages (TAMs), regulating their localization and retention within hypoxic tumor areas (13). This led us to question whether SEMA3F might also play a critical role in regulating neutrophil migration and thus the innate immune response to tissue injury. Neutrophil clearance from inflamed sites is regulated by onset of apoptosis (14), expulsion, and as more recently described, reverse migration away from tissues (15–17). The possibility of active signals retaining neutrophils within tissues has been less explored and we hypothesized that in addition to influencing neutrophil recruitment to tissues, SEMA3F might also regulate neutrophil retention at the injury site. Here we demonstrate that inflammatory neutrophils express SEMA3F, which can directly regulate neutrophil persistence and function at the inflamed site. This response is a result of dynamic filamentous-actin (F-actin) disassembly specific to the neutrophil. This work highlights a mechanism by which an axon guidance factor can both be delivered by inflammatory cells and selectively regulate their retention at sites of inflammation.

## Results

### Inflammatory human neutrophils express SEMA3F and its coreceptor NRP2.

**Figure 1:**
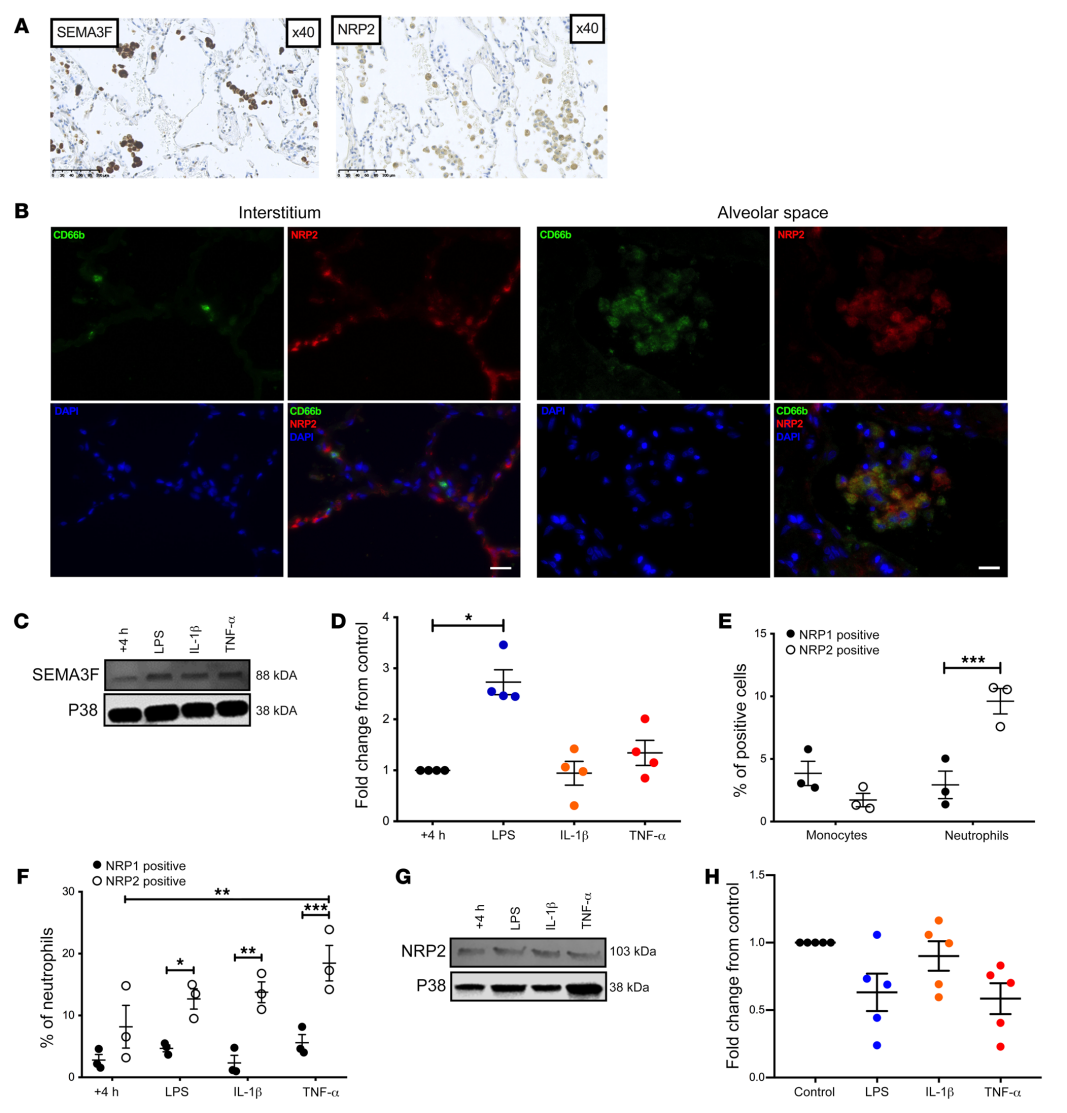
Inflammatory human neutrophils express SEMA3F and its coreceptor NRP2. (**A** and **B**) Lung sections taken at time of tumor resection from nontumor regions of patients with moderate severity COPD were stained for SEMA3F or NRP 2 (**A**), or a combination of CD66b (green), NRP2 (red), and DAPI (blue) (**B**). Images taken at ×40 magnification. Scale bars: 100 μm in **A**, 20 μm in **B**. Human blood neutrophil SEMA3F protein expression following 4 hours culture ex vivo was assessed by Western blot (**C**), and fold change to unstimulated control was determined by densitometry normalized to P38 (**D**). The percentage of blood monocytes (CD66b^–^, CD14/49D^+^) and neutrophils (CD66b^+^) expressing NRP1 and NRP2 was determined in freshly isolated cells (**E**) and following ex vivo culture for 4 hours by flow cytometry in control and stimulated conditions (**F**). Data are mean ± SEM, with individual data points (*n* = 3–5) from independent experiments. Human blood neutrophil NRP2 protein expression following 4 hours culture ex vivo was assessed by Western blot (**G**) and fold change to unstimulated control was determined by densitometry normalized to P38 (**H**). Statistical analysis: 1-way ANOVA and Bonferroni’s post hoc tests (**D** and **H**) and 2-way ANOVA and Sidak’s post hoc tests (**E** and **F**) were performed. *P < 0.05; **P < 0.01; ***P < 0.001.

We have previously established that the *Sema3f* transcript is highly upregulated in recruited PHD3-deficient neutrophils (9). This result suggests that neutrophil *Sema3f* expression is increased in the inflammatory niche and may have a role in inflammation. We first explored SEMA3F expression in chronic obstructive pulmonary disease (COPD), a disease characterized by neutrophilic inflammation. In tissue sections of surgically resected COPD lung, we identified staining for SEMA3F, and the coreceptor neuropilin 2 (NRP2) (Figure 1A), with NRP2 localized to the recruited CD66b^+^ myeloid cell populations (Figure 1B). Ex vivo, human peripheral blood neutrophils increased SEMA3F protein expression in response to stimulation with the proinflammatory mediator LPS at 4 hours but not at 12 hours (Figure 1, C and D and Supplemental Figure 1A; see complete unedited blots in the supplemental material available online with this article; https://doi.org/10.1172/JCI130834DS1). Equivalent levels of secreted SEMA3F were found between these treatment groups (Supplemental Figure 1B). In parallel, we assayed surface expression of SEMA3F receptors NRP1 and NRP2. Neutrophils, in contrast to circulating monocytes, preferentially expressed NRP2 rather than NRP1 (Figure 1, E and F). Ex vivo, total NRP2 protein expression was not inducible above untreated levels at 4 and 12 hours (Figure 1, G and H, and Supplemental Figure 1C). Naive and LPS monocytes, in contrast, failed to demonstrate expression of either SEMA3F or NRP2 (Supplemental Figure 1, D and E).

### Loss of sema3f accelerates inflammation resolution.

**Figure 2:**
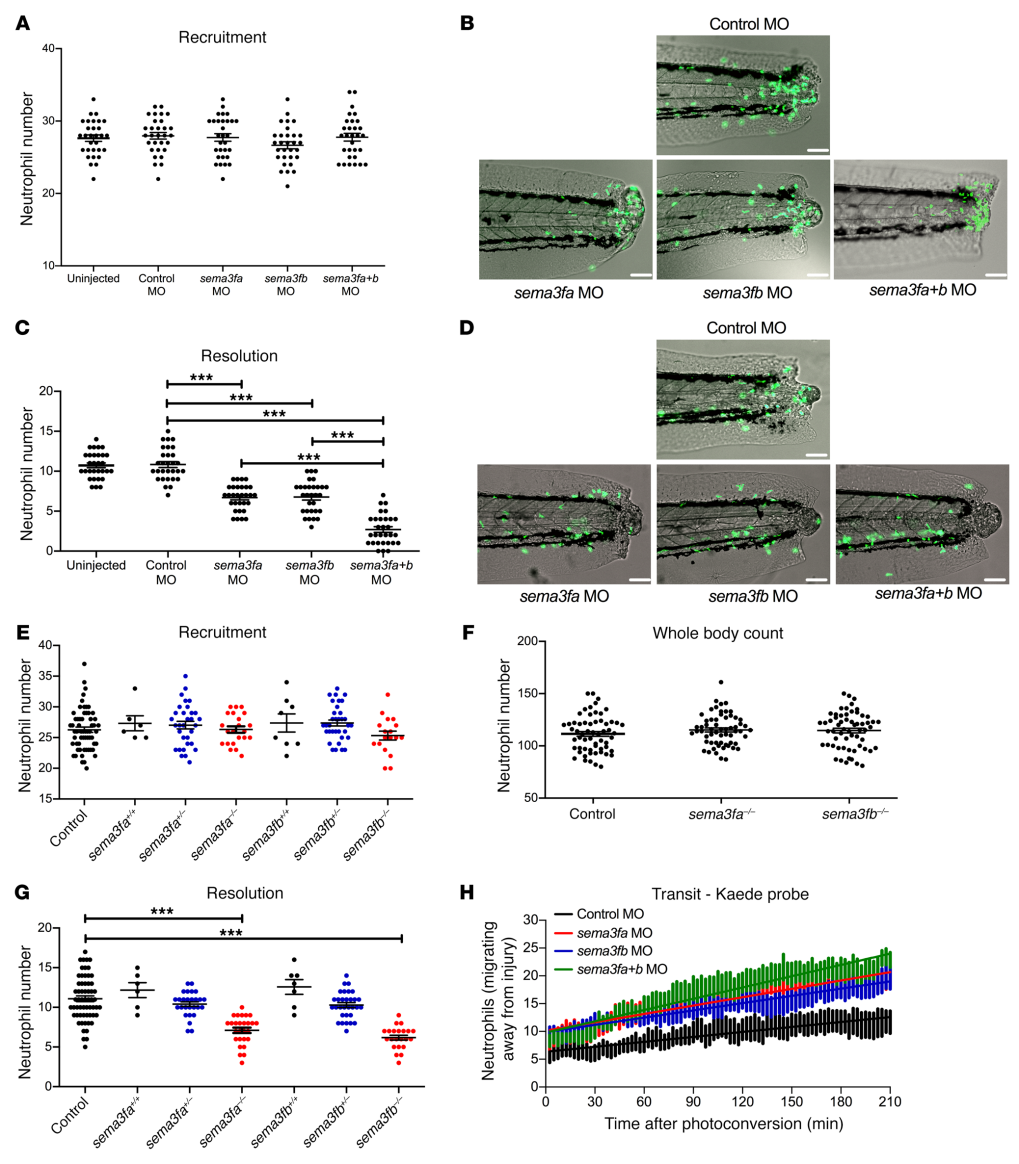
Knockdown of semaphorin 3F by MO injection or by TALEN-induced mutation accelerates resolution of neutrophilic inflammation in zebrafish. (**A**–**D**) *sema3fa* and/or *sema3fb* MO (1 nL of 0.5 mM) was injected into 1-cell-stage zebrafish mpx:GFP embryos, with 1 nL of 0.5 mM control MO used as a negative control. Tail fin transection was performed at 2 dpf, and neutrophils were counted at 6 hpi and 24 hpi. (**A**) Neutrophil counts at the 6 hpi time point with (**B**) overlaid fluorescence and bright-field photomicrographs (recruitment). (**C**) Neutrophil counts at the 24 hpi time point with (**D**) overlaid fluorescence and bright-field photomicrographs (resolution). Scale bars: 60 μm (**B** and **D**). Data are mean ± SEM, with individual data points (*n* = 30) from 3 independent experiments. (**E**–**G**) *sema3fa-* or *sema3fb*-mutated F1 fish were incrossed and compared with mpx:GFP fish. Tail fin transection was performed at 2 dpf, and (**E**) neutrophils were counted at 6 hpi. Data are mean ± SEM, with individual data points (*n* = 30) from 4 independent experiments. (**F**) Whole-body total neutrophil numbers were counted at 3 dpf. Data are mean ± SEM, with individual data points (*n* = 60) from 4 independent experiments. (**G**) Tail fin transection was performed at 2 dpf and neutrophils were counted at 24 hpi. Data are mean ± SEM, with individual data points (*n* = 5–60) from 4 independent experiments. (**H**) *sema3fa* and or *sema3fb* MOs (1 nL of 0.5 mM) were injected into 1-cell-stage zebrafish mpx:kaede embryos, and tail fin transection was performed at 2 dpf. Neutrophils at 6 hpi were recruited to the wound and photoconverted, and red fluorescence neutrophils were tracked for 3.5 hours. Data are from 3 independent experiments (*n* = 9). Statistical analysis was by 1-way ANOVA and Bonferroni’s post hoc test. ***P < 0.001.

To understand the consequences of SEMA3F expression for tissue inflammation, we turned to a zebrafish model of inflammation resolution, in which transgenically labeled neutrophils can be followed in transparent larvae during the recruitment and resolution phases of inflammation following tissue injury (18). Consistent with the idea that Sema3f impacts neutrophil function in this model, analysis of the single-cell RNAseq resource from zebrafish hematopoietic cells reveals that neutrophils express the *nrp2* coreceptor (http://www.sanger.ac.uk/science/tools/basicz). We therefore tested the effects of genetic ablation of *sema3f* by morpholino-modified (MO-modified) antisense oligonucleotide knockdown of both zebrafish homologs of human *SEMA3F* (*sema3fa* and *sema3fb*) (refs. 18, 19 and Supplemental Figure 2A). While knockdown of *sema3fa* or *sema3fb* alone or in combination using both translation- and splice-blocking MOs had no effect on neutrophil recruitment (Figure 2, A and B, and Supplemental Figure 2B), disruption of either *sema3fa* or *sema3fb* resulted in dramatically accelerated inflammation resolution. Either *sema3fa* MO or *sema3fb* MO injection alone resulted in fewer neutrophils being retained at the injury site 24 hours after injury (Figure 2, C and D, and Supplemental Figure 2C). Furthermore, an additive effect of *sema3fa* and *sema3fb* knockdown was observed (Figure 2, C and D). Importantly, knockdown of *sema3fa* or *sema3fb* had no effect on levels of neutrophil apoptosis during inflammation resolution in zebrafish or on whole-body neutrophil counts (Supplemental Figure 3).

To further confirm our knockdown experiments, we generated mutant zebrafish on an *mpx*:GFP background, in which stop codons were introduced into the *sema3fa* and *sema3fb* genes using genome editing with transcription activator–like effector nucleases (TALENs) (Supplemental Figure 4). Following tail fin injury, fluorescent neutrophil numbers at the inflammatory site were counted at 6 and 24 hours in progeny from an incross of heterozygotes of either *sema3fa* or *sema3fb*. Fish were subsequently genotyped and the data grouped by genotype. Neutrophil recruitment was independent of *sema3fa* or *sema3fb* genotype (Figure 2E), confirming our observations with MO knockdown. There were no significant changes in whole-body neutrophil counts in either mutant (Figure 2F). Inflammation resolution was, however, greatly accelerated in homozygous mutants of *sema3fa* or *sema3fb* (Figure 2G), recapitulating the morphant phenotype. Thus, SEMA3F is necessary for normal neutrophil retention at inflammatory sites in zebrafish. To better understand the dynamics of neutrophil egress from the injured site, we quantified the ability of neutrophils to reverse migrate following tail injury using the neutrophil-specific, photoconvertible Kaede reporter transgenic larvae (20). Photoconversion of neutrophils recruited to the wound area from green to red enabled the course and location of recruited neutrophils to be mapped over time. Injection of transgenic Kaede larvae with *sema3fa* or *sema3fb* MOs resulted in increased neutrophil reverse migration during inflammation resolution, with an additive effect seen when *sema3fa* and *sema3fb* MOs were injected together (Figure 2H).

### Neutrophil-specific loss of Sema3f results in more rapid neutrophil recruitment to and clearance from the lungs, with retained antimicrobial capacity.

**Figure 3:**
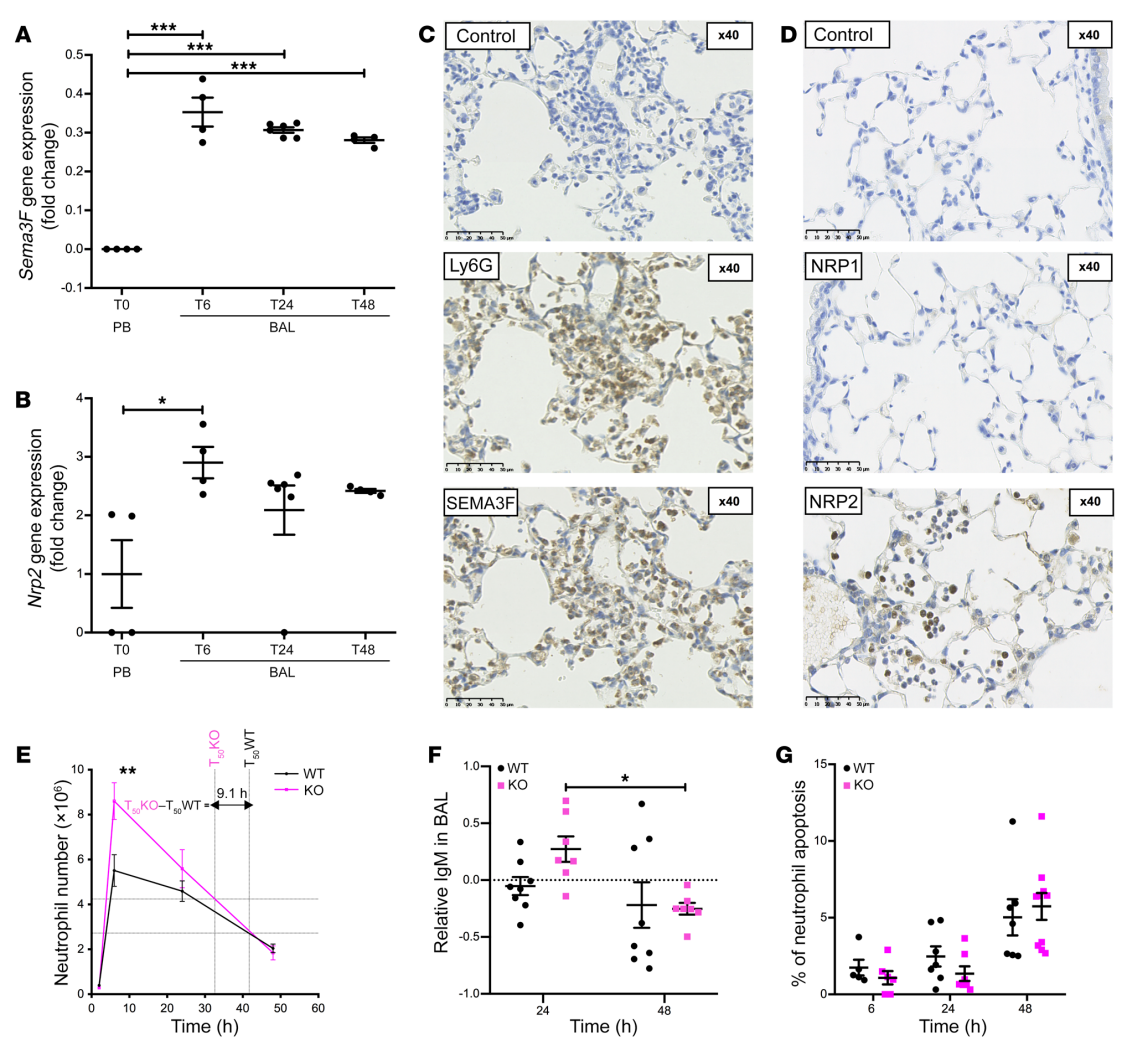
Neutrophil-specific loss of *Sema3f* results in more rapid neutrophil recruitment to and clearance from the lungs in a murine acute lung injury model. (**A** and **B**) Fold change in *Sema3f* and *Nrp2* gene expression following acute lung injury with LPS. Mice were sacrificed at 6, 24, and 48 hours after instillation. BAL neutrophils were collected and cDNA was extracted. TaqMan analysis of cDNA was performed with data normalized to murine *Actb* gene expression. Data are mean ± SEM of fold change compared with peripheral blood neutrophils (T0 PB) from 2 individual experiments (*n* = 4–6). An acute lung injury was induced by intratracheal LPS instillation, mice were sacrificed at 24 hours, and lung sections were stained for expression of the Ly6G neutrophil marker and SEMA3F (**C**), NRP1, and NRP2 (**D**). Scale bars: 50 μm. (**E** and **F**) *Sema3f^fl/fl^Mrp8Cre*^–/–^ (WT) and *Sema3f^fl/fl^Mrp8Cre^+/–^* (KO) mice were challenged with LPS, sacrificed at 2, 6, 24, and 48 hours, and BAL fluid was obtained. Cell counts were performed by hemocytometer and the differential cell count was established by cytospins. Time to 50% reduction in peak neutrophil number was calculated individually for each genotype (T_50_) (**E**). BAL fluid IgM content was measured by ELISA. Data are shown as log-transformed fold change from WT (**F**). Apoptosis was assessed by morphology, with data as mean ± SEM (**G**) from 3 individual experiments (*n* = 6–12). Statistical analysis was by 1-way ANOVA and Bonferroni’s post hoc test (**A** and **B**) and 2-way ANOVA with Sidak’s post hoc test (**E**–**G**). *P < 0.05; **P < 0.01; ***P < 0.001.

To further explore the consequence of neutrophil expression of SEMA3F for inflammation resolution in a mammalian system, we turned to a well-established murine model of LPS-induced acute lung injury. In response to LPS challenge, SEMA3F is released into the airways (Supplemental Figure 5A), with airspace neutrophils expressing *Sema3f* transcript and SEMA3F protein over an extended time course (Figure 3, A and C, and Supplemental Figure 5B). Neutrophils also expressed the obligate coreceptor *Nrp2* at both mRNA and protein levels (Figure 3, B and D), with 10% of airspace neutrophils demonstrating surface expression of NRP2 24 hours after LPS administration, in contrast to the alveolar macrophage population (Supplemental Figure 5C). NRP1 was not expressed (Figure 3D), replicating the preferential expression of NRP2 observed in human neutrophils (Figure 1, B, E, and F). Given the expression of SEMA3F by inflammatory neutrophils and the proresolution consequence of nonselective SEMA3F loss, we questioned whether neutrophil-specific downregulation of *Sema3f* was the key determinant for neutrophil egress from the lung. We therefore crossed *Mrp8 Cre* with *Sema3f^fl/fl^* lines to generate a transgenic mouse with *Mrp8*-driven neutrophil-specific knockdown of *Sema3f* (*Sema3f^fl/fl^Mrp8Cre^+/–^*) (Supplemental Figure 6, A–E). Following LPS challenge, *Sema3f^fl/fl^Mrp8Cre^+/–^* (KO) mice exhibited both a greater influx and more rapid rate of clearance of these cells (Figure 3E) than *Sema3f^fl/fl^Mrp8Cre^–/–^* WT littermate controls in keeping with more rapid transit of neutrophils through the lung compartment and loss from the airspace when they lack *Sema3f*. The resolution of neutrophilic inflammation occurred 9.1 hours earlier in the KO mice compared with WT mice, where resolution is defined as the time taken to reach a 50% reduction in the neutrophil number from peak counts. This was matched with a significant reduction in bronchoalveolar lavage (BAL) IgM levels between 24 and 48 hours in the *Sema3f*-deficient mice (Figure 3F). Importantly, at all time points shown, there was no observed difference in the percentage of neutrophils undergoing apoptosis between the genotypes (Figure 3G).

**Figure 4:**
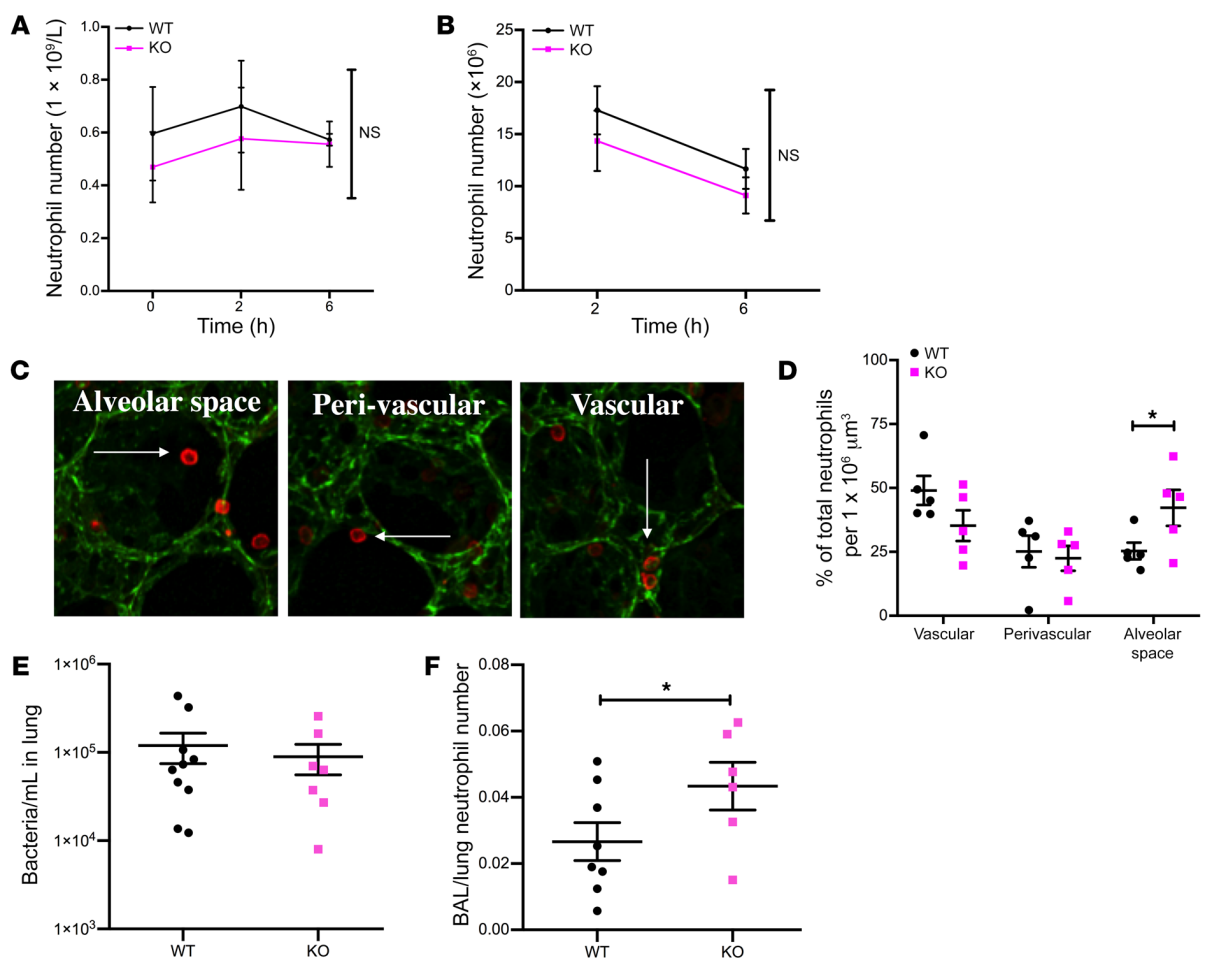
Neutrophil-driven deletion of *Sema3f* favors a selective allocation of neutrophils in the alveolar space while retaining antimicrobial capacity. (**A** and **B**) *Sema3f^fl/fl^Mrp8Cre*^–/–^ (WT) and *Sema3f^fl/fl^Mrp8Cre^+/–^* (KO) mice were challenged with nebulized LPS and sacrificed at 0, 2, and 6 hours following LPS challenge. Blood and lung tissues were harvested with lung digest for Ly6G staining (neutrophil number). In a parallel series of experiments, lungs were instilled with agarose gel at 6 hours, then fixed and stained with the endothelial marker CD31 (green) and the neutrophil marker S100A9 (red). Lungs were imaged by confocal microscopy (Zeiss LSM 880 with Airyscan) with 3D reconstruction and neutrophil position relative to the blood vessels assigned, using Imaris software version 9.1 (neutrophil, white arrows) (**C**). Percentage of total neutrophils per 10^6^ μm^3^ lung tissue is shown, with a minimum of 190 neutrophils quantified per mouse (**D**). Data are mean ± SEM from 3 individual experiments (*n* = 3–5). (**E** and **F**) WT and KO mice were challenged with intratracheal instillation of *S*. *pneumoniae*, and lung bacterial counts (**E**) and BAL and lung neutrophil counts (**F**) were undertaken 14 hours after challenge. Data are mean ± SEM from 2 individual experiments (*n* = 6–10). Statistical analysis was by 2-way ANOVA with Sidak’s post hoc test (**A**, **B**, and **D**) and Mann-Whitney (**E**) and 1-tailed unpaired *t* test (**F**). *P < 0.05.

To define how neutrophil-specific loss of *Sema3f* regulated airway neutrophil counts in the face of preserved apoptosis rates, we examined peripheral blood (Figure 4A and Supplemental Figure 6F) and whole lung neutrophil counts following lung digest (Figure 4B and Supplemental Figure 6G). We observed equivalent circulating and tissue neutrophil counts among genotypes up to 6 hours after LPS challenge and therefore tested whether differences in airway neutrophil counts observed reflected differential neutrophil transit among intravascular, perivascular, and alveolar compartments. Using 3D reconstruction of fixed lung slices we compared neutrophil localization in lungs harvested from WT and KO mice (Figure 4C). In keeping with more rapid transit through the lung tissue, a greater proportion of neutrophils were identified in the alveolar compartment of mice deficient in neutrophil *Sema3f* at 6 hours (Figure 4D and Supplemental Figure 6H), with equivalent numbers in the blood (Figure 4A) and lung tissue (Figure 4B and Supplemental Figure 6I), and clearance by 48 hours (Supplemental Figure 6H).

To delineate whether the inflammatory response of *Sema3f^fl/fl^Mrp8Cre^+/–^* was sufficient to control a fulminant infection, mice were challenged with intratracheal high-dose serotype 2 *Streptococcus pneumoniae*. Mice lacking *Sema3f* in the neutrophil compartment were able to mount an effective antimicrobial response with equivalent temperature and sickness scores (Supplemental Figure 7, A and B) and lung CFU counts (Figure 4E). In keeping with the LPS response, neutrophils transited more rapidly from the circulation (Supplemental Figure 7C) through the lung into the airspace compartment in the absence of *Sema3f* (Figure 4F).

### Upregulated sema3f expression causes aberrant neutrophil retention in tissue.

**Figure 5:**
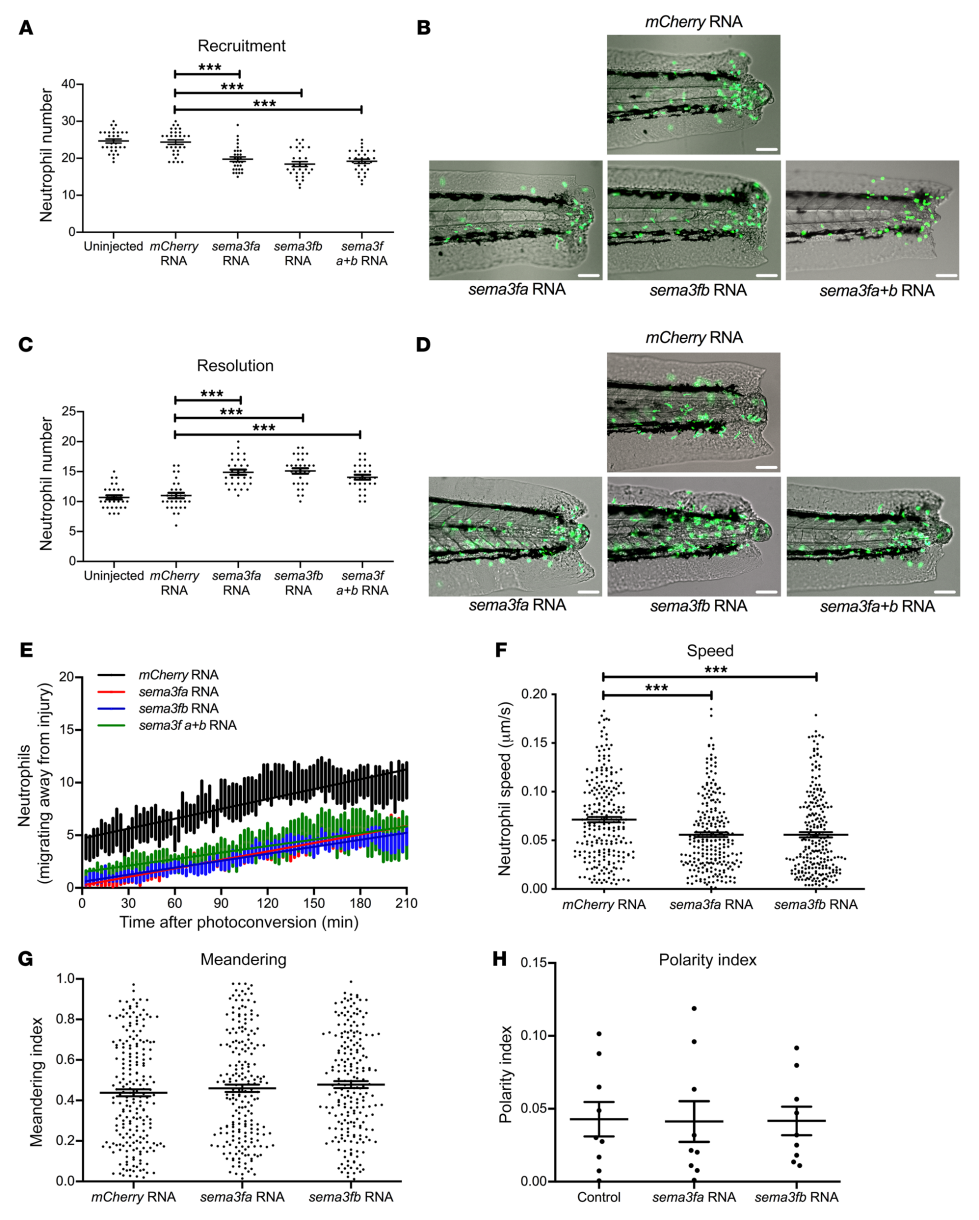
Overexpression of *sema3f* in a zebrafish model of inflammation delays neutrophil recruitment and resolution of the inflammatory response. (**A**–**D**) *sema3fa* or *sema3fb* RNA (50 ng/μL) was injected into 1-cell-stage zebrafish mpx:GFP embryos, with 50 ng/μL *mCherry* RNA used as a negative control. Tail fin transection was performed at 2 dpf, and neutrophils counted at 6 and 24 hpi. (**A**) Neutrophil counts at the 6 hpi time point with (**B**) overlaid fluorescence and bright-field photomicrographs (recruitment). (**C**) Neutrophil counts at the 24 hpi time point with (**D**) overlaid fluorescence and bright-field photomicrographs (resolution). Scale bars: 60 μm (**B** and **D**). Data are mean ± SEM with individual data points from 3 independent experiments (*n* = 30). (**E**) *sema3fa* and/or *sema3fb* RNA (50 ng/μL) were injected into 1-cell-stage zebrafish mpx:kaede embryos, and tail fin transection was performed at 2 dpf. Neutrophils recruited to the wound at 6 hpi were photoconverted, and red fluorescent neutrophils were tracked for 3.5 hours. Data are mean ± SEM from 3 independent experiments (*n* = 9). (**F** and **G**) *sema3fa* or *sema3fb* RNA (50 ng/μL) was injected into 1-cell-stage zebrafish mpx:GFP embryos, with 50 ng/μL *mCherry* RNA used for control. Tail fin transection was performed at 2 dpf. Neutrophil movement was tracked over 1 hour by time-lapse microscopy during the recruitment phase of inflammation (1–2 hpi) and speed of neutrophil migration (**F**) and meandering index (displacement/path length) (**G**) were determined. Data are mean ± SEM from 3 independent experiments (each point represents a single neutrophil) (*n* = 15). (**H**) *sema3fa* or *sema3fb* RNA (50 ng/μL) was injected into 1-cell-stage Tg(lyz:PHAkt-EGFP) embryos with noninjected controls, tail fin transection was performed at 2 dpf, and polarity indices were calculated for neutrophils recruited to the tail region. Data are mean ± SEM from a single experiment (*n* = 8). Statistical analysis was by 1-way ANOVA and Bonferroni’s post hoc test. ***P < 0.001.

Having shown that genetic ablation of *Sema3f* in vivo leads to premature neutrophil release from inflammatory sites, we sought evidence that SEMA3F is sufficient to restrain neutrophils’ migratory behavior during inflammation. Zebrafish *sema3fa* and *sema3fb* were cloned from cDNA and capped mRNA for each was transcribed in vitro then microinjected into fertilized eggs. Overexpression of *sema3fa* or *sema3fb* was confirmed by in situ hybridization (Supplemental Figure 8). Overexpression of either *sema3fa* or *sema3fb* resulted in a significant reduction in neutrophil recruitment to the injury site compared with larvae in which *mCherry* control mRNA was injected or to uninjected controls (Figure 5, A and B). When followed over time, neutrophils in larvae injected with mRNA for either *sema3fa* or *sema3fb* showed delayed clearance of neutrophils from the injury site when compared with uninjected and *mCherry* injected controls (Figure 5, C and D). The magnitude of the response was equivalent for both *sema3fa* and *sema3fb* with no additive effect observed, suggesting that either *sema3fa* or *sema3fb* could act to retain neutrophils at the inflammatory site. No effects on levels of neutrophil apoptosis during inflammation resolution or whole-body neutrophil numbers were observed (Supplemental Figure 9). In keeping with the importance of SEMA3F signaling in retaining neutrophils at inflammatory sites, injection of either *sema3fa* or *sema3fb* RNA led to a reduced number of photoconverted neutrophils moving away from the site of tail transection (Figure 5E). This was paralleled with a reduction in speed of migration (Figure 5F) but equivalent path straightness (meandering index) (Figure 5G) when tracking neutrophil movement over 1 hour during the recruitment phase of inflammation. Using a reporter zebrafish line that indicates intracellular PI3kinase activity by recruitment of a GFP-AKT pleckstrin homology (PH) domain fusion protein to phosphoinositide products on the neutrophil cell membrane, we observed that SEMA3F overexpression did not affect the ability of the neutrophils to generate a leading edge, as judged by activation of PI3K at the plasma membrane (Figure 5H and ref. 3).

### Exogenous SEMA3F retains recruited neutrophils at the injury site in a murine model of acute lung injury.

**Figure 6:**
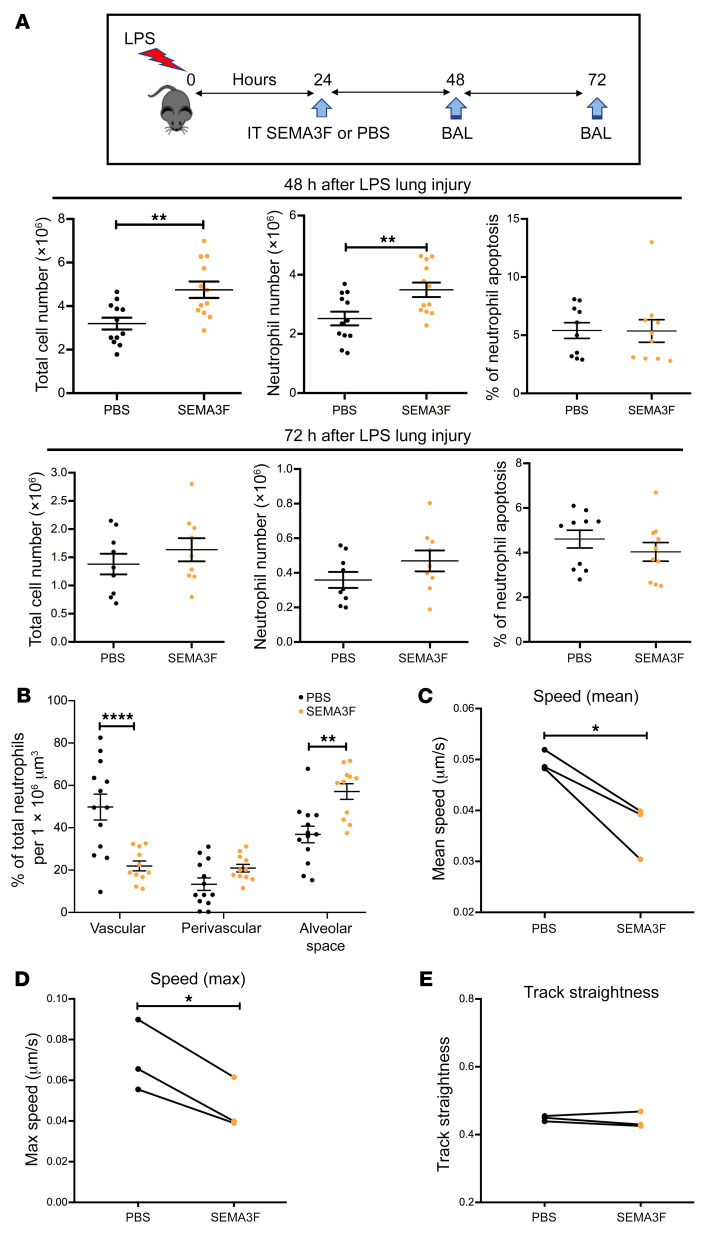
Exogenous SEMA3F retains recruited neutrophils at the injury site in a murine model of acute lung injury. (**A** and **B**) Intratracheal (IT) recombinant SEMA3F (1 μM) was administered to C57BL/6 mice 24 hours after nebulized LPS challenge or PBS. Mice were then sacrificed at 48 and 72 hours and BAL was performed with differential apoptosis cell/neutrophil counts (**A**), or lungs were retained for fixed lung slice imaging (**B**). Lungs harvested for lung imaging were instilled with agarose gel, and fixed and stained with the endothelial marker CD31 (green) and the neutrophil marker S100A9 (red). Lungs were imaged by confocal microscopy (Zeiss LSM 880 with Airyscan) with 3D reconstruction and neutrophil position relative to the blood vessels was assigned using Imaris software version 9.1, with at least 80 neutrophils quantified per mouse. Data are mean ± SEM with individual data points from 4 independent experiments (*n* = 12). (**C**–**E**) Naive Catchup (IVM-RED;Lifeact-GFP) mice were sacrificed and lungs were instilled with agarose gel, precision sliced, and imaged by confocal microscopy for 90 minutes with addition of SEMA3F or PBS vehicle control at 30 minutes. Following treatment, neutrophil mean speed (**C**), maximum speed (**D**), and track straightness (directionality) (**E**) were measured and analyzed for 60 minutes using Imaris software version 9.1. Data are from 3 independent experiments (*n* = 3). Statistical analysis was by 2-way ANOVA and Sidak’s post hoc test (**A** and **B**) or paired *t* test (**C**–**E**). *P < 0.05; **P < 0.01; ****P < 0.0001.

To investigate the effects of exogenous SEMA3F on inflammation resolution in the murine model of acute lung injury, 24 hours after LPS challenge, SEMA3F (1 μM) was instilled into the trachea of mice and total cell and neutrophil differential counts were performed at 48 and 72 hours (Figure 6A). More neutrophils were recovered from the 48-hour BAL samples of mice receiving exogenous SEMA3F than from PBS control (Figure 6A). Again, this occurred despite equivalent apoptosis counts between SEMA3F-treated and SEMA3F-naive mice. Quantifying the localization of neutrophils in fixed lung slices as before, this increase in BAL neutrophil counts was paralleled by a decrease in the vascular space and an increase in neutrophils within the alveolar compartment, the compartment in which neutrophil exposure to exogenous SEMA3F occurred (Figure 6B). Given the observed difference in speed of neutrophil movement in the tail of zebrafish larvae, we tested whether exogenous SEMA3F could also act to alter the speed of neutrophil movement in the lung. Imaging live lung slices from Ly6G-driven fluorescent reporter mice (21, 22) in which murine neutrophils express the reporter tdTomato, we quantified mean neutrophil speed (Figure 6C), maximum speed (Figure 6D), and track straightness (track straightness was determined by dividing the distance between first and last position by the length of the tracks) (Figure 6E). Live lung slice cultures harvested from naive mice were subject to in vitro stimulation with SEMA3F or vehicle control at 30 minutes and neutrophil behavior was recorded for 60 minutes. Baseline neutrophil behavior during the first 30 minutes was similar between paired slices (Supplemental Figure 10). SEMA3F significantly reduced both mean and maximum neutrophil speed (Figure 6, C and D) while neutrophil track straightness remained unchanged (Figure 6E). Thus, neutrophils move more slowly when exposed to SEMA3F and as a consequence are retained at sites of inflammation.

### Human neutrophil treatment with exogenous SEMA3F blocks chemotaxis while preserving phagocytosis and respiratory burst activity.

**Figure 7:**
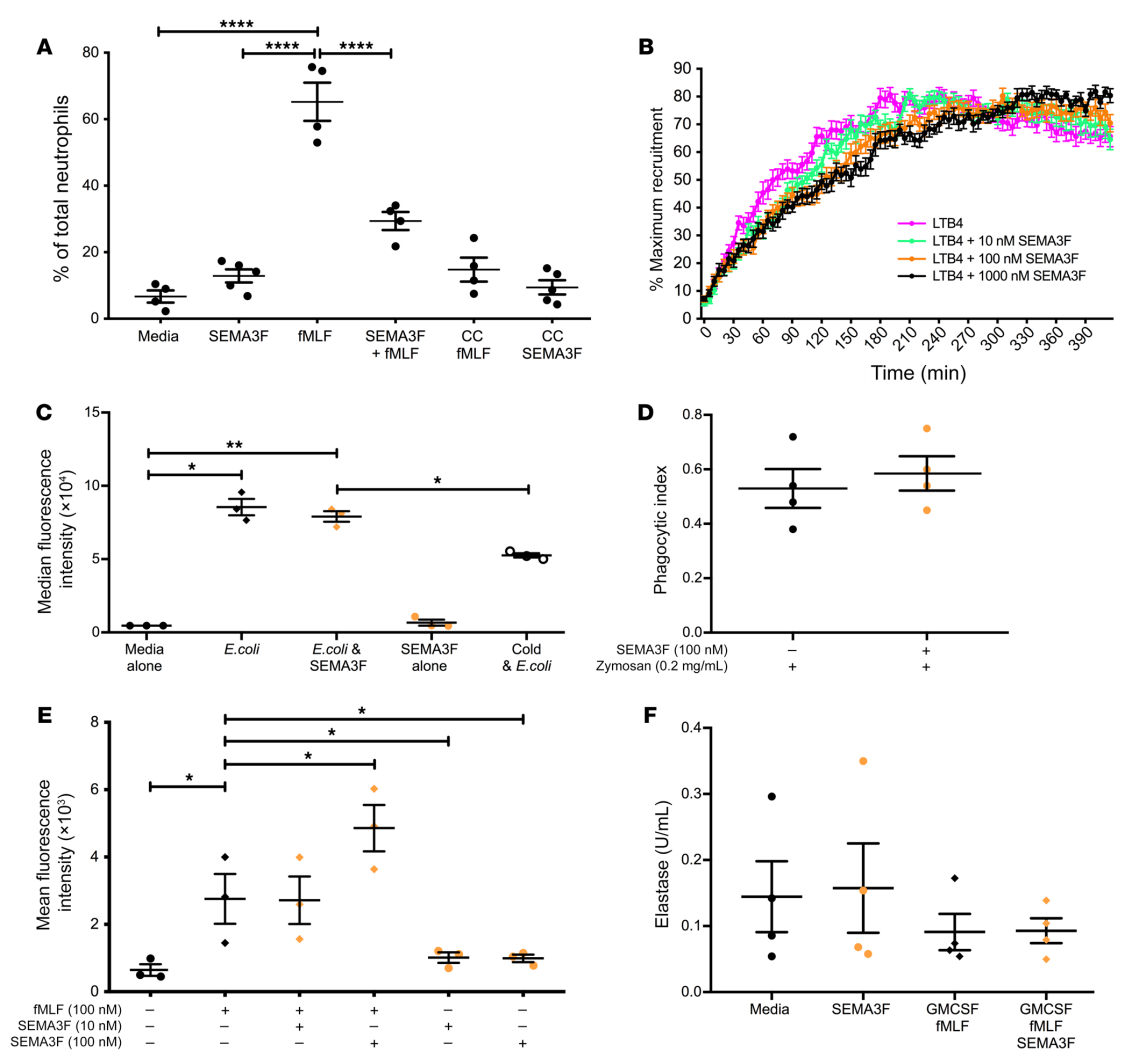
Neutrophil treatment with exogenous SEMA3F blocks chemotactic responses while preserving phagocytic capacity and respiratory burst functions. (**A**–**F**) Isolated peripheral blood neutrophils from healthy volunteers were incubated with recombinant SEMA3F (0–100 nM), and functional assays were performed. (**A** and **B**) Chemotactic behavior of neutrophils to fMLF (0–100 nM) and LTB4 were measured by Boyden chamber, CC chemokinesis control (**A**), and microfluidic chip assay (**B**). (**C**) Neutrophils were incubated with Alex 488 *E*. *coli* and phagocytic uptake was determined by flow cytometry, with adhesion excluded by 4°C control. (**D**) Phagocytic indices were calculated by cytospin following neutrophil culture with opsonized Zymosan particles for 30 minutes. (**E**) ROS generation was determined following a 1 hour preincubation with SEMA3F and treatment with fMLF for 30 minutes. (**F**) Neutrophil release of elastase was measured by fluorimetric assay following pretreatment with 1 hour of SEMA3F, 30 minutes of GM-CSF (10 ng/mL), and 10 minutes of fMLF (100 nM). All data are mean ± SEM with individual data points from independent experiments (*n* = 3–6). Statistical analysis was by 1-way ANOVA and Sidak’s post hoc test (**A**, **B**, **E**, and **F**) or paired *t* test (**C**–**E**). *P < 0.05; **P < 0.01; ****P < 0.0001.

To more broadly assess the ability of SEMA3F to regulate key human neutrophil functions, human peripheral blood neutrophils were cultured ex vivo in the presence of recombinant SEMA3F. Using a Transwell assay, we demonstrated that although SEMA3F is not a chemoattractant itself, incubation with SEMA3F inhibits ex vivo human neutrophils migrating toward the chemoattractant formyl-methionyl-leucyl phenylalanine (fMLF) (Figure 7A). To further investigate the effect of SEMA3F on neutrophil migration, we used a microfluidic chip assay providing bidirectional real-time data with a stable LTB4 chemoattractant gradient (23). SEMA3F preincubation resulted in a significant dose-dependent reduction in recruitment (Figure 7B). Moreover, after reaching the inflection point (where maximal recruitment is achieved) the highest dose of SEMA3F, 1000 nM, was able to retain neutrophils within the chamber. In this setting, SEMA3F treatment of human peripheral blood neutrophils ex vivo did not alter neutrophil surface expression of key adhesion receptors CD11b or L selectin (Supplemental Figure 11, A and B). Phagocytosis is also an important cytoskeletal neutrophil function that, like migration, is regulated by cell shape and actin dynamics. SEMA3F treatment did not inhibit phagocytosis of either bacteria (*E*. *coli*) or yeast (Zymosan) by neutrophils (Figure 7, C and D). Surprisingly, SEMA3F augments the capacity of neutrophils to mount a respiratory burst response to fMLF (Figure 7E) while preserving neutrophil degranulation determined by measure of extracellular elastase activity in neutrophil supernatants (Figure 7F).

### SEMA3F promotes neutrophil rounding and F-actin disassembly.

**Figure 8:**
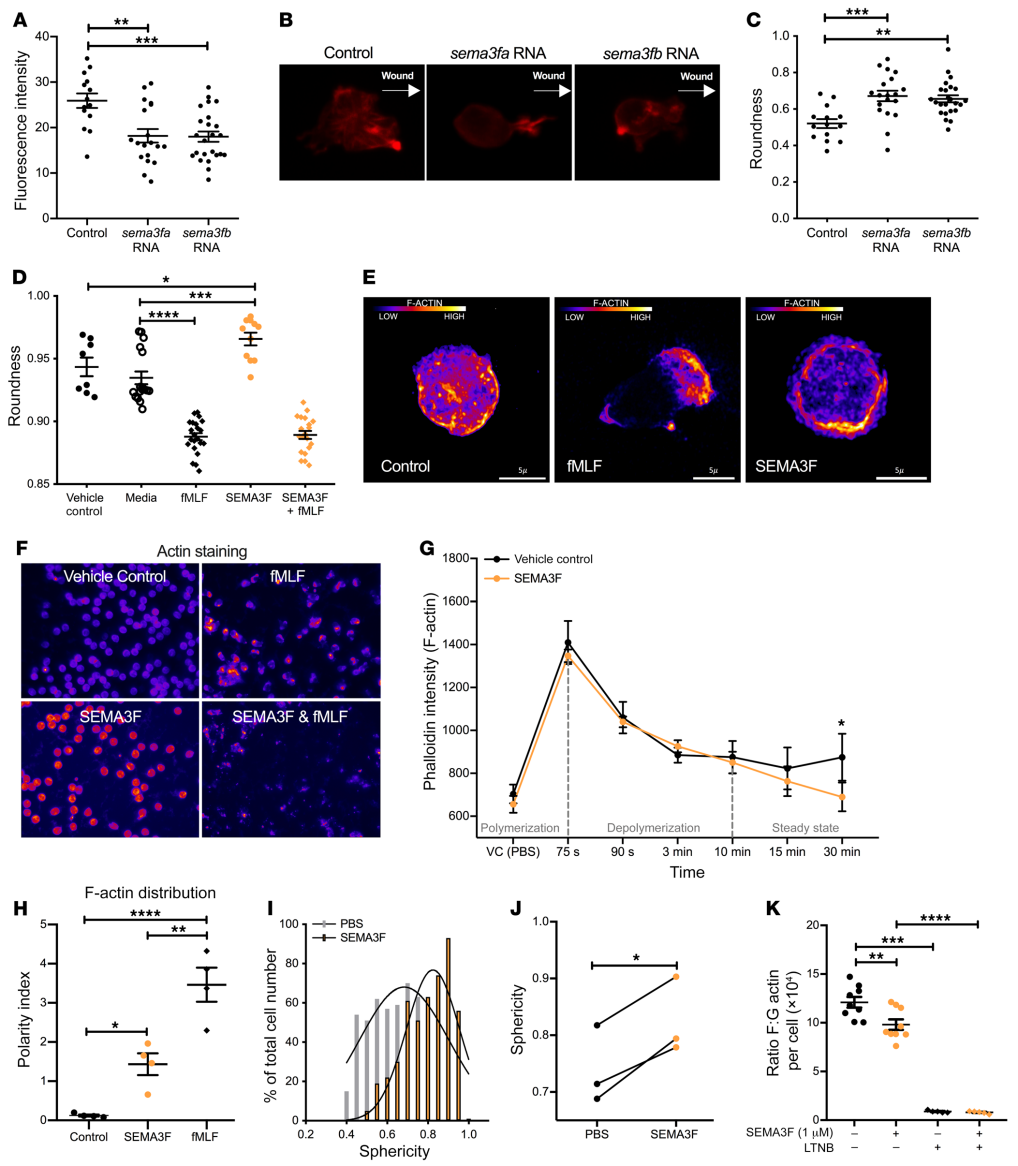
SEMA3F promotes neutrophil rounding and F-actin disassembly. (**A**–**C**) *sema3fa* or *sema3fb* RNA (50 ng/μL) was injected into 1-cell-stage Tg(mpx:LifeActRuby) embryos, tail fin transection was performed at 2 dpf, and neutrophil fluorescence intensities were calculated (**A**). Representative images (**B**). White arrows indicate the direction to the wound edge. Roundness scores were calculated for neutrophils recruited to the injury (**C**). Data are mean ± SEM from an single experiment (*n* = 9). (**D**–**G**) Human blood neutrophils were pretreated with PBS or 100 nM SEMA3F before stimulating with PBS or 100 nM fMLF. Following DAPI/cell mask/phalloidin staining, neutrophil rounding was quantified by high-content widefield microscopy (1 = perfect sphere), with more than 1000 cells measured per condition (**D**). Pixel intensity for phalloidin was obtained using confocal microscopy (×100 objective), scale bars: 5 μm (**E**). F-actin cell content was quantified with more than 1000 cells measured per condition (**F** and **G**) and distribution was used to calculate a polarity index (**H**). Data are mean ± SEM from 4 independent experiments (*n* = 4–22). (**I**–**K**) Intratracheal SEMA3F (1 μM) was administered to C57BL/6 mice 24 hours after nebulized LPS or PBS, and lung tissue (**I** and **J**) or BAL fluid (**K**) was collected at 48 hours. Lungs were instilled with agarose gel, fixed and stained with the endothelial CD31 and the neutrophil marker S100A9, and imaged by confocal microscopy. The percentage of neutrophils of each sphericity (**I**) and the mean sphericity of neutrophils (**J**) was defined. F/G actin ratios per cell were calculated from fluorescence intensities following staining with phalloidin (F-actin) and DNAse 1 (G-actin), with a latrunculin B (LTNB) negative control (**K**). Data are mean ± SEM from 2 independent experiments (*n* = 9) (**I**–**K**). Statistical analysis was by 1-way ANOVA and Bonferroni’s post hoc test (**A**–**D** and **H**), with Sidak-Holm multiple comparison posttest (**K**) and paired *t* test (**G**) performed for time points 15 to 30 minutes during the steady state of F-actin turnover, and unpaired *t* test (**I**–**J**). *P < 0.05, **P < 0.01, ***P < 0.001, ****P < 0.0001.

**Figure 9:**
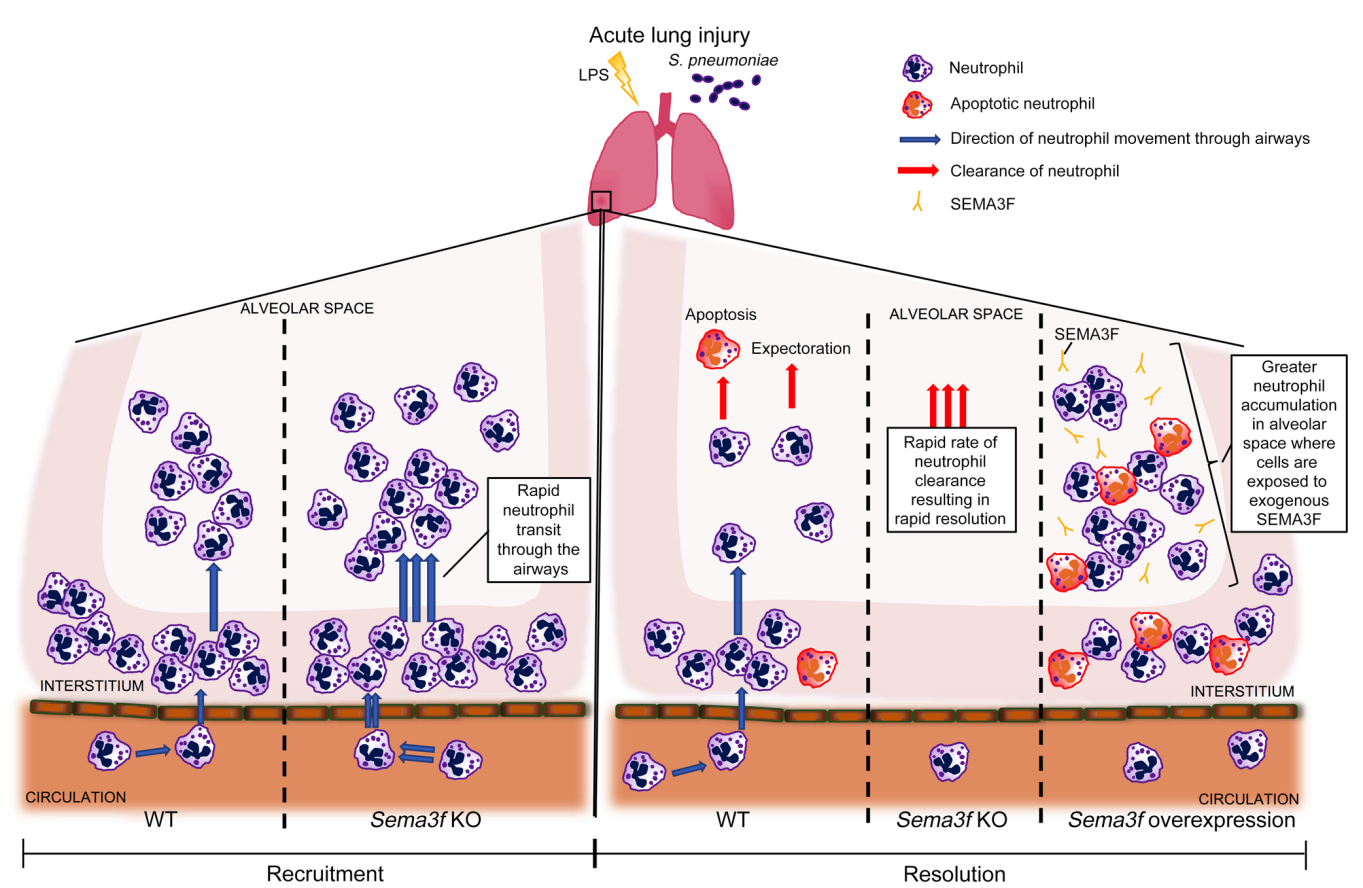
A summary of the migratory movement of neutrophils in *Sema3f* knockdown and overexpression models of acute lung injury. Neutrophil-specific KO of *Sema3f* increased neutrophil transit through the airways and led to more rapid clearance of neutrophils from the alveolar space, resulting in faster resolution. In contrast, overexpression of *Sema3f* in the airspaces caused neutrophil retention and delayed inflammation resolution.

To investigate the mechanism by which SEMA3F slows neutrophil migration in vivo, we returned to the zebrafish model system. We focused on the potential of SEMA3F to mediate its effect by alterations in F-actin polymerization in light of the observations that during axonal growth cone collapse, the class 3 semaphorin SEMA3A induces F-actin reorganization, and the reports that F-actin polarization is important for neutrophil motility (24–28). Using a transgenic zebrafish line in which F-actin fluoresces red (4, 29) we observed that *sema3fa* and *sema3fb* RNA overexpression resulted in a reduction in overall F-actin levels (Figure 8, A and B) and an increase in neutrophil rounding (Figure 8, B and C). High content wide-field imaging revealed an increase in neutrophil rounding in human peripheral blood neutrophils when treated in vitro with SEMA3F and was recapitulated using Airyscan confocal imaging shown in the representative images (Figure 8, D and E). This neutrophil rounding was associated with a reduction in end steady state F-actin levels following stimulation of neutrophils with fMLF (Figure 8, F and G). To address whether changes in F-actin levels were consequent, in part, upon changes in actin turnover, we quantified F-actin localization following neutrophil treatment with SEMA3F. In keeping with defective turnover, SEMA3F induced aberrant distribution of F-actin filaments (Figure 8H and Supplemental Figure 11C). To further test the hypothesis that in neutrophils SEMA3F promotes F-actin disassembly, neutrophil rounding, and retention within the airways, we studied fixed lung slices harvested from WT mice challenged with nebulized LPS and treated with SEMA3F or control PBS ex vivo. SEMA3F treatment increased both the percentage of neutrophils that are highly round and the mean sphericity of neutrophil populations within the lung tissue (Figure 8, I and J). To assess alterations in F-actin polymerization, we quantified the ratio of F/G actin within each airway neutrophil. In keeping with loss of F-actin content, mice exposed to SEMA3F had significantly lower F/G actin ratios per neutrophil than SEMA3F-naive controls (Figure 8K). There is a dynamic balance between neutrophil migration between and retention within the different compartments of the lung, and consequence for effective immunity and inflammation resolution (Figure 9).

## Discussion

To date, effective therapeutic strategies that target persistent neutrophilic inflammation remain an important unmet clinical need. In this work, we identify a mechanism by which inflammatory cells can deliver a neutrophil-specific retention signal to the injury site in the form of a secreted axon guidance molecule, SEMA3F. Initial phenotyping revealed, both acutely in a murine LPS model of lung injury and chronically in lung sections from patients with COPD, that recruited myeloid cells express the class 3 semaphorin SEMA3F and its high-affinity coreceptor NRP2. With evidence that CD66b^+^ cells express NRP2 in the interstitium and alveolar space, and that LPS upregulates SEMA3F protein expression, these data together speak of a dynamic SEMA3F/NRP2 axis within the inflammatory neutrophil compartment. While neutrophil production and release of preformed and newly synthesized factors that modify outcomes of key neutrophil responses (including pathogen clearance, recruitment, and survival) are well established, the expression of molecules that function to retain neutrophils within the injury site represents a mechanism through which the longevity of the inflammatory response may be regulated.

Using zebrafish models, we showed that SEMA3F regulates neutrophil migration speed, but not directionality, with consequences for both neutrophil recruitment and tissue retention. This was recapitulated in a murine model of lung injury, where SEMA3F was seen to control neutrophil recruitment and retention at the injury site. The relevance of this response to human neutrophil behavior was supported by our observation that while exogenous SEMA3F does not act as a chemoattractant, it profoundly inhibits neutrophil chemotaxis to fMLF and LTB4 and is an active retention signal. The ability of axon guidance molecules to regulate leukocyte migration has been previously described both in lymphocytes and HL-60 cells, with the neuronal repellent Slit inhibiting chemotactic responses in vitro to stromal cell–derived factor 1 (SDF-1a) (30), and recently, the regulation of trans-endothelial neutrophil migration by HIF-dependent endothelial expression of SEMA7A has also been investigated (31). These observations are further supported by the finding that T lymphocyte transmigration can be modified by gene silencing of the collapsin response mediator protein 2, a previously characterized effector of semaphorin-induced growth-cone collapse (32). SEMA3E, another class 3 semaphorin, signals independently of NRP coreceptor expression, unlike SEMA3F (33). More recently, Movassagh et al. identified that SEMA3E was a chemorepellent and modulator of neutrophil migration in the context of chronic allergen-driven lung inflammation, in a whole animal *Sema3e^–/–^* murine model (34). Our work both extends the role of class 3 semaphorins in regulating neutrophil movement to include SEMA3F and raises the concept of active neutrophil retention within the inflamed site. With induction of SEMA3F observed in neutrophils recruited in response to tissue injury, sterile inflammation, and pathogen challenge, this raises the interesting possibility that a number of divergent signals, including for example damage associated molecular patterns (DAMPs) and pathogen associated molecular patterns (PAMPs), may act in concert to actively retain neutrophils within the inflammatory niche. Within the lung tissue, retention of neutrophils has important consequences for inflammation resolution, as evidenced by the increase in neutrophil clearance in mice lacking SEMA3F within the neutrophil compartment. In the lung, expectoration of neutrophils from the airspace represents a major mechanism of clearance, in addition to neutrophil apoptosis both in the airspaces and lung interstitium. While perivascular reverse migration of neutrophils is observed, there are currently no data to suggest that neutrophils can return to either the vasculature or lymphatics once they have entered the airways. Thus, the transition times of neutrophils between the different lung compartments will dominate rates of inflammation resolution in the setting of differential SEMA3F expression, where effects on neutrophil apoptosis are not observed.

Within axons, actin rearrangement is observed following activation of semaphorin signalling pathways. In our experiments, we also observed that SEMA3F promotes neutrophil rounding in association with F-actin disassembly and persistence within the airspaces. Taken together, these data suggest a mechanism by which neutrophil expression of SEMA3F selectively regulates neutrophil retention within the inflammatory site as a consequence of promoting F-actin disassembly and suppressed neutrophil migratory speeds. Future work to explore the mechanisms by which neutrophil SEMA3F expression regulates neutrophil retention within the tissue and whether this is a cell autonomous mechanism or dependent on the presence of other immune cell populations (e.g., alveolar macrophages) will be of interest. Importantly, this occurs in the face of preserved leading edge PI3kinase activity, preserved directional migration, and critically preserved neutrophil effector functions. This is in keeping with SEMA3F acting downstream of the PH domain proteins, with F-actin disassembly and aberrant F-actin distribution, and turnover selectively affecting neutrophil retention in the face of preserved antimicrobial effector function. Thus we propose that SEMA3F acts as a break in neutrophil movement among the different vascular, interstitium, and airspace compartments within the lung.

Finally, when considering the therapeutic utility of targeting SEMA3F to promote inflammation resolution, the consequence of *Sema3f* loss within the neutrophil compartment on effective antimicrobial responses was explored in vivo. Importantly, neutrophils deficient in *Sema3f* were able to mount an antimicrobial response that was sufficient to control bacterial numbers where animals were exposed to a bacteremic model of streptococcal pneumonia. With a dissociation between the consequences of *Sema3f* loss on neutrophil migratory speed versus respiratory burst generation and bacterial clearance, an important future direction of this work will be to dissect the downstream mechanisms by which neutrophils retain key effector functions in the face of aberrant F-actin turnover and distribution.

In summary, our work aims to elucidate how neutrophils respond to signals within an inflamed microenvironment that induce them to persist. We identify SEMA3F as a neutrophil retention signal with effects conserved across a number of different species (fish, mouse, and human). These observations have important implications for inflammation resolution and tissue injury and are of interest given both the current lack of effective therapeutic strategies for treating neutrophil inflammation and the potential to selectively target the neutrophil retention response.

## Methods

### Immunohistochemistry.

Murine paraffin-embedded blocks were prepared from lungs fixed via the trachea with 10% buffered formalin at 20 cm H_2_O. Serial sections were stained with anti-Ly6G clone 1A8 (ab25377, Abcam) and the following polyclonal antibodies: anti-SEMA3F (SAB2700501, Sigma-Aldrich), NRP1 (AF3870, R&D Systems), NRP2 (H300, Santa Cruz Biotechnology), or an isotype control following deparaffinization. Lung sections from patients with COPD undergoing resection for suspected lung tumor were stained with anti-SEMA3F, NRP2, or isotype control, and visualized with DAB or stained with anti-CD66b (555723, BD Biosciences), anti-NRP2 (HPA054974, Atlas Antibodies), and DAPI (422801, Sigma-Aldrich) with TSA plus system amplification (NEL744B001KT, Perkin Elmer) and autofluorescence quenching with TrueView (Vector, SP-8400).

### Isolation and culture of human blood neutrophils.

Human peripheral blood neutrophils were isolated from whole blood using dextran sedimentation and discontinuous plasma-Percoll gradients. Cells were cultured 5 × 10^6^ cells/mL in RPMI 1640 supplemented with 10% FCS and 1% penicillin/streptomycin at 37°C in a humidified incubator with 5% supplemental CO_2_ in the presence or absence of LPS from *Escherichia coli* (10 ng/mL, Enzo Life Sciences), IL-1b (100 ng/mL, AMS Biotechnology), TNFa (100 ng/mL, AMS Biotechnology), or LTB4 (100 nM, Cayman Chemical) for 4 to 12 hours.

### Protein expression.

Lysates were prepared using sonication in a Bioruptor*PLUS* iced water bath (Diagenode Europe SA) and added to lysis buffer. Following SDS-PAGE protein separation (7.5% gels were appropriate for all proteins studied), transfer of proteins to a PVDF membrane was performed using a Bio-Rad Mini Trans-Blot cell at 100 V for 90 minutes. Intercept (PBS) blocking buffer (Li-Cor) was used to block blots and dilute primary and secondary antibodies. Primary antibodies were directed against SEMA3F (1:100; LS-C135015, LifeSpan Biosciences) and NRP2 (1:100; HPA054974, Atlas Antibodies), and P38 (1:2000; ab197348, Abcam) was used as a loading control. Blots used for the detection of SEMA3F were incubated with horseradish peroxidase–conjugated (HRP-conjugated) anti-rabbit (1:2000; P0448, Dako), and blots used for the detection of NRP2 were incubated with IRDye800CW-conjugated anti-rabbit (1:2000; 926-32213, Li-Cor). Signals were detected using an Odyssey FC Imaging System (Li-Cor) and protein expression was quantified by scanning densitometry and normalized to the expression of P38. Enzyme-linked immunosorbent assay (ELISA) was performed according to the manufacturer’s protocol to quantify SEMA3F levels (Cloud-Clone Corporation) in supernatants of human blood neutrophils following 4 hours culture ex vivo with inflammatory stimuli, IgM levels (Abcam) in BAL fluid from mice either after 24 or 48 hours after nebulization with LPS (Sigma-Aldrich, 1 mg), or after 14 hours post-intratracheal instillation of 10^7^ *S*. *pneumoniae* D39 type 2 strain. Flow cytometry was performed to analyze surface expression of L selectin and CD11b by using anti–L selectin [PE] (BD Pharmingen) and anti-CD11b [PE] (BD Pharmingen), respectively.

### Isolation and RNA quantification of human and murine neutrophils.

Human peripheral blood neutrophils were isolated as described above, with murine peripheral blood neutrophils isolated from WT C57BL6 mice by negative magnetic selection (Easysep; STEMCELL Technologies) and inflammatory neutrophils were recovered from BAL fluid following challenge with 1 mg nebulized LPS. RNA was extracted using the mirVana total RNA isolation protocol (Ambion). Samples were treated with DNAse (Ambion) and random hexamer cDNA was synthesized by reverse transcription. TaqMan commercially available primer probe sets were obtained from Applied Biosystems for the target assay (*Sema3f*) and the endogenous control assay (*actb*) (Applied Biosystems).

### Quantification of murine neutrophil numbers and NRP2 surface expression during LPS-mediated acute lung injury and intratracheal S. pneumoniae infection.

Murine venipuncture was performed directly following humane killing and full blood counts were conducted using the Coulter counter method by the clinical pathology service at the Royal Dick Veterinary School, Edinburgh, Scotland. For *S*. *pneumoniae*–infected mice, blood was harvested from abdominal aorta after humane killing and nucleated blood cells were counted using an automated cell counter (Biorad) after red blood cell lysis (BioLegend). Total viable neutrophil counts were obtained after blocking unspecific staining with Fc block (BioLegend) and using live/dead aqua dye (Applied Biosystems) in combination with the following antibodies (BioLegend): anti-CD45 [AF700] (clone 30-F11), anti-CD11b [BV450] (clone M1/70), and anti-Ly6G [PE] (1A8). For total lung counts or after infection, lung digest was performed with Collagenase V (Sigma-Aldrich), Collagenase D (Roche), DNAse I (Roche), and Dispase (Gibco). Following red cell lysis (Sigma-Aldrich), viable cell counts were performed using live/dead aqua dye (Applied Biosystems). Immune cell populations were identified after Fc block (BioLegend) using the following markers (all from BioLegend): anti-Siglec F [PE-CF594] (clone S17007L ), anti-CD11b [BV450] (clone M1/70), anti-Ly6C [FITC] (clone HK1.4), anti-Ly6G [PE] (clone 1A8), anti-MHC II [PerCP-Cy5.5] (clone C068C2), anti-CD11c [PE-Cy7] (clone N418), anti-CD64 [APC] (clone X54-5/7.1), and anti-CD45 [AF700] (clone 30-F11). BAL fluid was harvested from mice after LPS or after infection and total nucleated cell counts were obtained either with hemocytometer or NucleoCounter (Chemometec). Total viable counts for the different immune populations were obtained by using either cytospins or the same markers used for lung tissue. Flow cytometry was performed to analyze NRP2 surface expression by using anti-NRP2 [APC] (clone 257103; R&D Systems).

### Bacterial viable counts.

Fourteen hours after infection with 10^7^ serotype 2, *S*. *pneumoniae* left lung was homogenized in sterile conditions by using a tissue homogenizer (Bullet Blender) and lysing tubes (Precellys). Ten-fold serial dilutions were plated into blood agar plates, grown overnight at 37°C and 5% CO_2_, and CFU counts determined.

### Zebrafish lines.

Adult zebrafish were maintained on a 14-hour-light/10-hour-dark cycle at 28°C in United Kingdom Home Office–approved facilities in the Bateson Centre aquaria at the University of Sheffield. The following neutrophil-specific fluorescent zebrafish lines were used: *Tg(mpx*:*GFP)i114* (called *mpx:*GFP in the text for clarity), *Tg(mpx:GAL4)sh267;Tg(UAS:kaede)s1999t*, *Tg(lyz:PHAkt-EGFP)i277, Tg(mpx:LifeActRuby)sh429*, and macrophage-specific fluorescent zebrafish line *Tg(fms:GAL4.VP16)i186;Tg(UAS:nfsB.mCherry)c624*. Transcription activator–like effector nuclease (TALEN) technology was used to induce frameshift mutations in *sema3fa* and *sema3fb* genes. Fertilized eggs were injected with RNAs coding for heterodimers of a left and right subunit containing a C-terminal Fok1 nuclease domain and an N-terminal site-specific DNA binding domain, specifically targeting *sema3fa* or *sema3fb* using the tool on http://talent.cac.cornell.edu/node/add/talen-old, with TALE repeats designed to flank the Mwol (*sema3a*) and BsII (*sema3b*) restriction endonuclease restriction site of exon 8 as previously described (15, 16). Germline mutation was verified by genomic PCR from larval pools of the F1 or F2 zebrafish using 5′-GGCGACGAGGTGGTCGTTGG-3′ and 5′-CATCGCGCAATGCACTGAC-3′ primers and appropriate restriction enzyme digest.

### sema3f knockdown and overexpression in the zebrafish models.

DNA was subcloned from plasmid constructs gifted by C. Moens (University of Washington, Seattle, Washington, USA) containing full-length zebrafish *sema3fa* and *sema3fb* in pCR4 vectors, RNA transcribed by mMessageMachine (Ambion) and microinjected into one-cell-stage embryos. All MOs were from Genetools. One-cell-stage embryos were microinjected with pre-mRNA translation blocking MO to *sema3fa* (17) or splice blocking MO targeting the exon3/5 boundary of *sema3fb,* 5′TATGAAGCGATACTCACGTTTGTGT3′. Efficacy of *sema3fb* knockdown was confirmed by RT-PCR (Supplemental Figure 2). The control MO was CCTCTTACCTCAGTTACAATTTATA.

### Inflammation assay in zebrafish.

Inflammatory responses were elicited in zebrafish embryos by tail transection as previously described (8). Neutrophils were counted at the site of transection at 6 and 24 hours after injury (hpi) using a fluorescence dissecting stereomicroscope (Leica Microsystems GmbH). Where possible, counting was performed blind to experimental conditions. For analysis of speed and meandering index of neutrophils toward the site of injury, embryos were mounted in 1% low melting point agarose (Sigma-Aldrich) containing 0.017% tricaine, and tracked over 1 hour using a 1394ORCA-ERA camera (Hamamatsu Photonics Inc.) on a TE2000-U Inverted Compound Fluorescence Microscope (Nikon). Tracking analysis was performed using Volocity 5 software (Improvision, Perkin Elmer).

### Analysis of neutrophil polarity and roundness in neutrophils.

*Tg(lyz:PHAkt-EGFP)i277* larvae were injured 3 days after fertilization (dpf), mounted in 1% low melting point agarose (Sigma-Aldrich) containing 0.017% tricaine, and imaged on an UltraVIEWVoX spinning disk confocal microscope (Perkin Elmer) with an inverted Olympus IX81 microscope. Neutrophils in the region between the site of injury and the posterior blood island were individually imaged in the 488 nm laser line with 20 *Z* slices. Using Image J software, a transecting line was drawn from the trailing edge to the leading edge and a fluorescence intensity plot generated. Polarity index was calculated by (log10a/b) × ([a + b]/c), where *a* represents the mean fluorescence intensity of the trailing edge of the cell, *b* represents the mean fluorescence intensity of the leading edge of the cell, and c represents the mean fluorescence intensity of the whole cell (20). In parallel, roundness was calculated using 4 × [area]/π × [major axis], where 0 represents a line and 1 a circle.

### Animals.

Mice were maintained in 12-hour-light/12-hour-dark cycles with free access to food and water. They were sex-, litter-, and age-matched into groups of 4 or 6 for experiments and then housed with their experimental littermates to avoid distress. Each mouse was given a numerical code and the genotype was revealed to the researcher following data analysis. MRP8-driven Cre (*Mrp8Cre*) targeted *Sema3f* deletions to neutrophil populations. *Sema3f^fl/fl^* mice were generated via the Medical Research Council (MRC) International Mouse Phenotyping Consortium (SEMA3F_HEPD0570_6_A04, allele type Tm1c) with animals backcrossed to a C57BL/6 background (35–37). Catchup (IVM-RED Lifeact-GFP) mice were used for in vivo neutrophil imaging studies (21, 22). Rectal temperatures were acquired, and sickness scores were undertaken by blinded observers as follows: 0, normal health; 1, lethargy and ruffled fur; and 2, severe lethargy, ruffled fur, and hunched back.

### Intratracheal instillation of exogenous SEMA3F.

Mice were anesthetized with an intraperitoneal injection of medetomidine (1 mg/kg) and ketamine (76 mg/kg), and then instilled with 50 μL PBS or 1 μM SEMA3F via the trachea, followed by recovery with 1 mg/kg atipamezole.

### Fixed and live murine lung slice imaging.

After humane killing, the trachea was cannulated and instilled with 2% (wt/vol in PBS) low melting point agarose gel (Life Technologies). Lungs were dissected en bloc, washed in PBS, and placed in either methanol-free 4% formaldehyde solution in PBS (made by diluting 16% formaldehyde, vol/vol; Thermo Fisher Scientific, Pierce, catalog 28908) for 2 hours (fixed lungs) or RPMI media (live lung culture), and 300 μm vibratome sections were generated (38). Fixed lung slices were incubated with unconjugated anti-S100A9 (HM1102, Hycult Biotech) and anti-CD31 (ab119341, Abcam), and secondary staining performed using Cy3 AffiniPure Donkey anti-rat IgG (712-165-150, Jackson ImmunoResearch Laboratories), goat anti-hamster IgG (H+L) Cross-Adsorbed Secondary Antibody, Alexa Fluor 488 (A-21110, Thermo Fisher Scientific) and DAPI. Lung slices were fixed and mounted using Mowiol with 2.5% DABCO (1,4-diazabicyclo-[2,2,2]-octane; Sigma-Aldrich). For live lung culture, slices were cultured in RPMI 1640 media in double glass-bottomed plastic chambers (Thermo Fisher Scientific). Slices were weighted with a steel washer overlying a tissue harp (Warner Instruments) and placed inside an environmentally controlled chamber. After 1 hour equilibration, Alexa Fluor 647 anti-mouse CD31 antibody (102415, BioLegend) and Alexa Fluor 488 anti-mouse S100A9 antibody (NBP2-47980AF488, Novus) were added directly to the media (38). Laser scanning confocal microscopy was performed using an inverted LSM 880 Airyscan Fast microscope (Carl Zeiss) and Airyscan processing was performed using default settings. Acquisition for both cultured and fixed slices used a Zeiss ×20 objective (0.8 NA, Plan Apochromat Air). During live lung imaging, preprogrammed automated time-lapse acquisition of cultured lung slices was used. Sequential alternating imaging of the control versus treated lung slice over the experiment approximated simultaneous recording. *Z*-stacks were acquired and projected using Imaris (Bitplane, Oxford Instruments) for analysis. For fixed slices, image stitching was performed using Zen black or blue software (Carl Zeiss) and represented an area of 1 mm × 1 mm of tissue, imaged at 1 time point/position. Image analysis was performed using IMARIS V 9.1 software (for examples of Imaris analysis, see Supplemental Videos 1 and 2).

### Neutrophil chemotaxis and phagocytosis functional assays.

Neutrophil chemotaxis to fMLF (0–100 nM) over 60 minutes was measured in the presence or absence of recombinant SEMA3F protein (10–100 nM) (R&D Systems) using 5-μm filter NeuroProbe ChemoTx microplates. For microfluidic chip assays, whole blood was applied to a stable LTB4 gradient (100 nM) and migration assessed using real-time light microscopy (21). To determine degree of phagocytosis, neutrophils were incubated with 0.2 mg/mL opsonized Zymosan A particles for 15 minutes at 37°C. Cytocentrifuge slides were then prepared and uptake of the yeast particles was assessed. The Phagocytic Index was calculated as follows: Phagocytic Index = (mean number of particles per neutrophil) × (percentage of neutrophils containing particles). Neutrophils were incubated with heat-inactivated fluorescein isothiocyanate–conjugated *E*. *coli* O55:B5 (Sigma-Aldrich) (MOI 1:1) for 30 minutes at 37°C. Cells were washed with ice-cold PBS, resuspended in FACS buffer, and analyzed by flow cytometry (BD FACSCalibur, BD Biosciences, Becton Dickinson Ltd.). Data analysis was performed with FlowJo software (version 9.0, Tree Star Inc.).

### Respiratory burst.

Human neutrophils (1 × 10^6^/mL) were cultured with recombinant SEMA3F protein (10 nM, 100 nM) (R&D Systems) for 1 hour before addition of 5 μM 2′7′-dichlorofluorescein diacetate (DCF) for 30 minutes. Cells were then stimulated with 100 nM fMLF for 30 minutes and FL1 geometric mean fluorescence was determined by flow cytometry.

### F-actin quantification and distribution.

Isolated peripheral blood neutrophils from healthy volunteers were incubated with recombinant SEMA3F (100 nM) and fMLF (100 nM). Neutrophils were fixed, permeabilized, stained with DAPI, and Alexa Fluor 488–conjugated phalloidin. Images were obtained using an Operetta High-Content Imaging System (Perkin Elmer) or LSM880 Airyscan Fast (Zeiss) and analysis of F-actin was performed by Harmony analysis software (Perkin Elmer).

### Statistics.

The data are expressed as mean ± SEM. Graph and curves were made using GraphPad Prism software versions 7 and 8. Mann-Whitney, unpaired, and paired Student’s *t* tests were used to compare 2 groups. One-way and 2-way ANOVA with Bonferroni’s, Dunnett’s, or Sidak’s post hoc test were used to compare different groups if the data followed a normal distribution and if the samples analyzed had the same genetic background. *P* values smaller than 0.05 were considered significant. The number of fish, mice, and donors used are listed in the figure legends.

### Study approval.

All participants gave written informed consent in accordance with the Declaration of Helsinki principles. Healthy human blood donation was obtained under the University of Edinburgh ethics protocol of the project entitled, *The Role of inflammation in Human Immunity*, AMREC reference 15-HV-013. This study was approved by the The Blood Resource Management Committee, Centre for Inflammation Research, Queen’s Medical Research Institute, Little France, Edinburgh; the Academic and Clinical Central Office for Research and Development (ACCORD) medical research ethics committee, AMREC (a joint office of the University of Edinburgh and National Health Service [NHS] Lothian). Human lung tissue was obtained by biopsy through the Edinburgh Tissue Bank, which was reviewed and approved by the Edinburgh Tissue Bank ethics committee and AMREC. The NHS Lothian Bio-Resources provided lung tissue from nontumor regions of lung following biopsy used for the immunohistochemistry in this project (application number SR451). All animal experiments were conducted in accordance with the Home Office Animals (Scientific Procedures) Act of 1986 with local ethical approval.

## Author contributions

TP, SE, MASG, LR, PC, ASM, DH, XLRI, LMC, JMM, FEE, TM, ERW, FM, AZ, JLC, and CL performed the research. TP, SE, MASG, LR, FVE, and PME interpreted the data. TP, SAR, AJWF, MKBW, and SRW designed the research and wrote the manuscript.

## Supplementary Material

Supplemental data

Supplemental Video 1

Supplemental Video 2

## Version 1 03/19/2020

In-Press Preview

## Version 2 05/18/2020

Electronic publication

## Version 3 06/01/2020

Print issue publication
