# Supplementary material for: Semaphorin 3F signaling actively retains neutrophils at sites of inflammation
Source: J Clin Invest. 2020 May 18;130(6):3221–37. doi: 10.1172/JCI130834 (PMC7259996; doi:10.1172/JCI130834)
Supplement: Supplemental data [file jci-130-130834-s455.pdf]

Supplemental Figure 1.

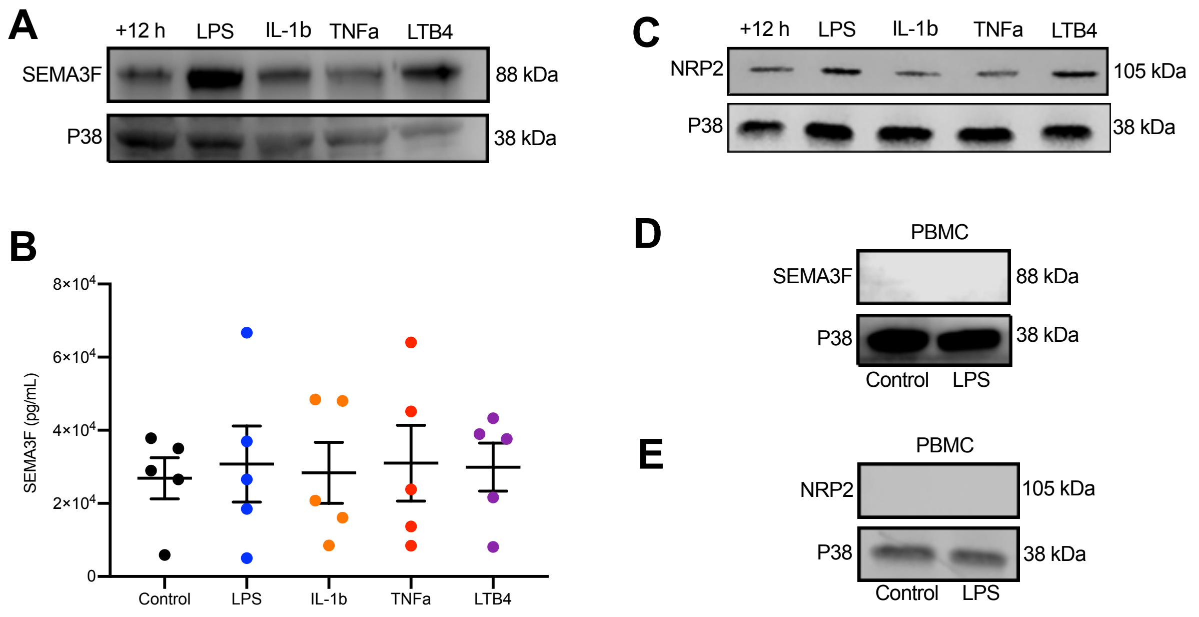

Supplemental Figure 1. Human neutrophils, but not monocytes express SEMA3F and its co-receptor NRP2. Human blood neutrophil SEMA3F protein expression following 12 h culture ex vivo was assessed by western blot (A), with secreted SEMA3F measured in cell culture supernatants at 4 h by ELISA (B). Concurrent expression of NRP2 was assessed by western blot (C). Human blood monocyte (PBMC) SEMA3F protein expression (D) and its co-receptor NRP2 (E) following 4 h culture ex vivo was assessed by western blot. Data shown are mean ± SEM, n=5 performed as five individual experiments. Statistical analysis: One-way ANOVA and Bonferroni's post-hoc test was performed.

Supplemental Figure 2.

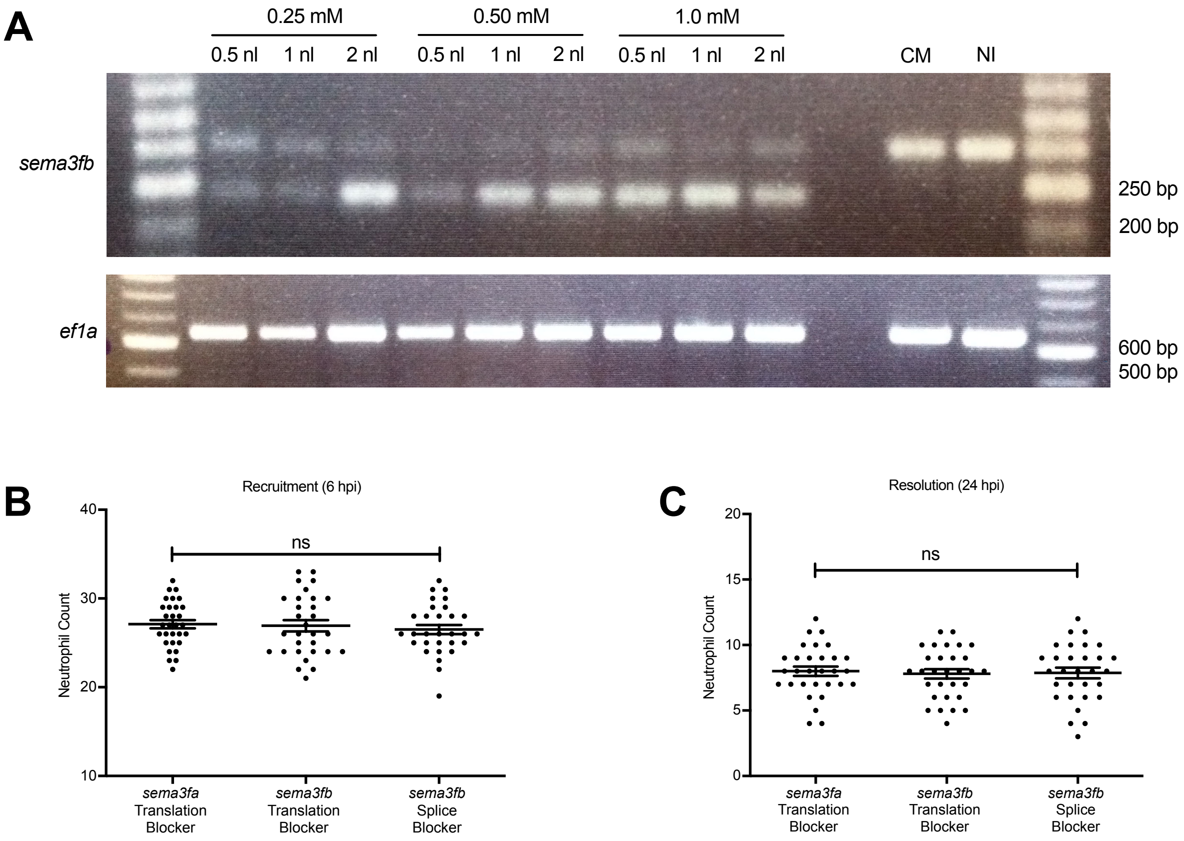

Supplemental Figure 2. Verification of genetic ablation of *sema3fb* by morpholino (MO) injection. (A) One-cell stage embryos were microinjected with a pre-*m*RNA splice blocking MO targeting the exon3/5 boundary and efficacy confirmed by PCR. The upper band delineates wild-type and lower band exon 4 deletion, with *ef1a* used as a loading control (CM control MO, NI non-injected control). (B, C) *sema3fa* or *sema3fb* translation blocking or *sema3fb* splice blocking MOs (1 nl of 0.5 mM) were injected into 1-cell stage zebrafish mpx:GFP embryos, tail fin transection performed at 2 days post fertilization (dpf), and neutrophils counted at 6 and 24 hours post injury (hpi) to allow comparison of MO efficacy. Data shown are mean ± SEM, n=30 performed as 3 individual experiments. Statistical analysis: One-way ANOVA and Bonferroni's post-hoc tests were performed.

Supplemental Figure 3.

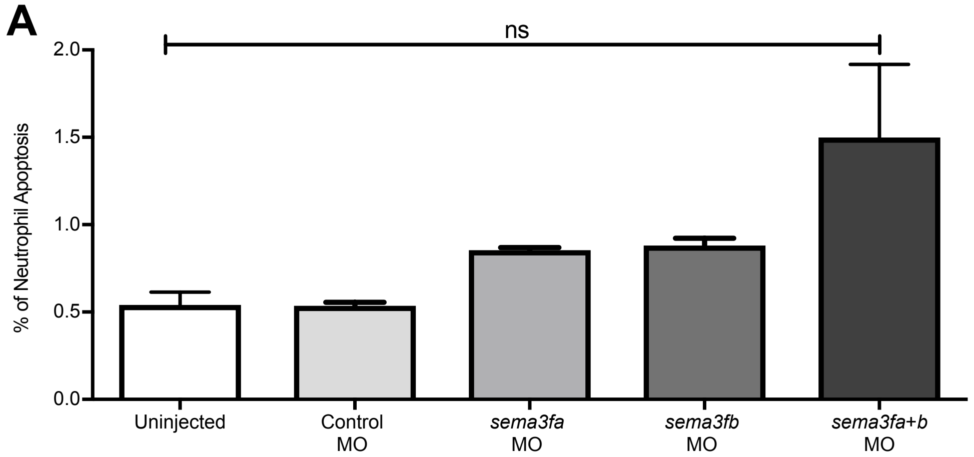

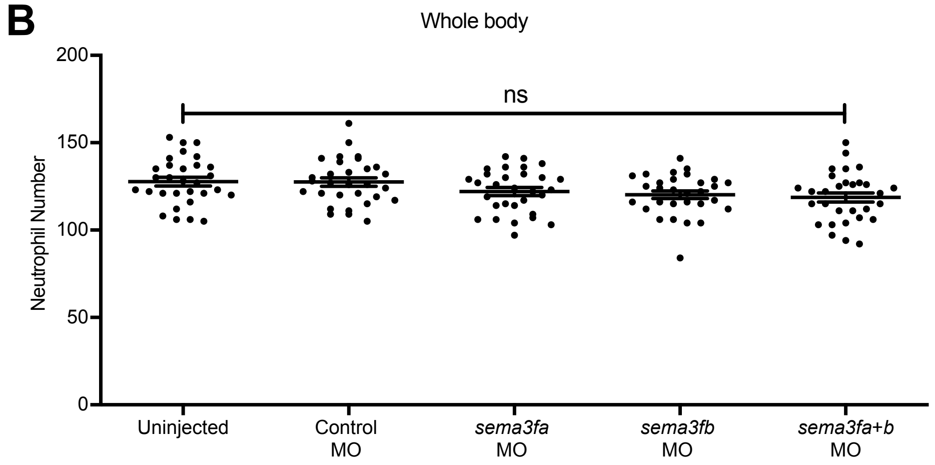

Supplemental Figure 3. Preserved apoptosis responses and total neutrophil counts with *sema3fa* and *sema3fb* morpholino (MO) injections. (A, B) *sema3fa* (translation blocking) or *sema3fb* (splice blocking) or *sema3fa+b* MOs were injected into 1-cell stage zebrafish mpx:GFP embryos. (A) Tail fin transection was performed at 2 dpf, and at 24 hpi neutrophils stained for terminal deoxynucleotidyltransferase-mediated dUTP nick end labeling and Tyramide signal amplification process for endogenous peroxidase activity. Data shown are mean ± SEM, n=180 performed as 3 independent experiments. (B) Whole body neutrophil counts were performed at 3 dpf. Data shown mean ± SEM, n=30 performed as 3 independent experiments. Statistical analysis: One-way ANOVA and Bonferroni's post-hoc tests were performed.

Supplemental Figure 4.

A

Semaphorin3Fa WT protein sequence
MDKVIMQGAGTLVLGTLLVSVFSAVSVHSSPLSSLPPASVPRVFLSFKELKSTGTAHHFAFLLNSTDYRILRMDEDHDRMY
VGSKDYILSLDLNDINKEPLIIHWPVAPQKRTECVLSGKDINGECGNFIRLIEPWNRTHLYVCGTGAYNPICTYVDRGQRAQ
GFLQDQMPRAGGRTSRAADPSASPEPYAPKEYIFRLEPGKVDSGKGKCPYDPKLNSVSALINGQLYAGVYIDFMGTDSAI
FRTLGKHTAMRTDQYNSRWLHDPTFVHAQLIPDSAEKNDDKLYFFFREKASEMGQTPMAQSRIGRICLNDDGGHCCLVN
KWSTFLKARLICSVTGSDGIETHFDELRDVYIQKTQDTKNPVIYGVFSVSGSVFKGSAVCVYSMADVRMVFNGPFAHKEG
PNYQWVAYQGKIPYPRPGTCPGGTFTPNMKSTKDYPDEVINFMRTHPTMFNAVYPVHKRPLVVRTNVDYEFTTITVDQVA
AADGNYEVLFLGTDRGTVQKVIVLPRDDLQTEELVLEEVEVFRVPTSITTMKISPKRQQLYVSSAVGVTHLALHRCDVYGE
ACADCCLARDPYCAWDGKSCSRYSANQKRRSRRQDVKYGNPIRQCRGYNSNANKNTLETVQYGVEGSTAFLECQARS
PHVSIKWHFQKENSDRRREIRSDGRVVRTEQGLLLRSLQLSDSGVYQCTSTEKNFKHTLVKLQLVVLSAHTVNSIQTESG
APAAPPLQSSAWTPSAEQYKDLLTILSQPEMGLINQYCQDYWQIGDDPMAHKKKDLKELQKDLRKPRNRRHHQEQSSMA
ET*

Semaphorin3Fa mutant protein sequence
MDKVIMQGAGTLVLGTLLVSVFSAVSVHSSPLSSLPPASVPRVFLSFKELKSTGTAHHFAFLLNSTDYRILRMDEDHDRMYVG
SKDYILSLDLNDINKEPLIIHWPVAPQKRTECVLSGKDINGECGNFIRLIEPWNRTHLYVCGTGAYNPICTYVDRGQRAQGFLQ
DQMPRAGGRTSRAADPSASPEPYAPKEYIFRLEPGKVDSGKGKCPYDPKLNSVSALINGQLYAGVYIDFMGTDSAIFRTWA
NTRQ*GPTSITPAGSTIQHLSMLSSSLIVQKRMTTSCISSSVRRPLRWVRLPWLNLGLAEFA*MMMEDTAVW*TSGAPS*RLG
SSALSPALMASRRISMSSGTCTSRRLKTQRILSSTASFPCPGLCLKAQQYVFIQWLMCAWSSMGLSLIKKAQTTNGLHTRAK
SHTLAQEHVQEGRLPPT*SLLKITQMR*STSCAHIPPCSMQFIQSTSAPWWYEPTWIMSSPPSLWTKWLQLMATMKFCS*EQ
TGEQFRK*LSCPEMIYRLKSWFWRRWRSSEFQLQSLL*RFLQNDNSCMSVRRWASHIWLCTDVMFTERPVPTAAWPETHT
VPGTESPALATQQTKKGGVEGKT*SMETL*GNAEATTRMPIRTHWRQCSME*REAQPFWSVRPDLLMCPSSGISRRRTATG
EERSVLTGVWFARNRASSCAPSSCLTAVFTSAPQLRRTSNTPLSNSSSWFSPPTQSTASRLKAAPRPPHPFSPAPGRPAQN
NTKTS*PSSANLRWASSTSTAKTTGR*GTTPWPTRRRTSRSSRRTCGSLEIGDTTRSRAAWLRH

B

Semaphorin3Fb WT protein sequence
MLLDSLWPVQVLLVLSVLTLVLGNEPQNAPRVFLSFKDLRSTGTAHYFSFLLNTSDYRILRMDEDHDRMYVGSKDYILSL
DLHDINREPLIIHWPVSPQRKTECVLSGKDTNGECGNFIRLIEPWNRTHLYICGTGAYNPVCTYVNRGRKPMTAMHLQM
PQTRGRASRAAEPETVFDETGLQEYVFHLEPGKEDSGKGKCPYDPKLNSVSALINGELYAGVYIDFMGTDSAIFRTLGK
NAVMRTDQYNSRWLNDPSFVHVHLIPDSGEKNDDKLYFFFREKSSEMGQSPKSQSRIGRICLNDDGGHCCLVNKWST
FLKARLICSVPGADGIETHFDELRDVYIQPTQDTKNPVIYGVFSVSGSVFKGSAVCVYSMADIRMVFNGPFAHKEGPNY
QWVAYTGKIPYPRPGTCPGGTFTPNMKSTKDYPDEVINFMRNHPTMYNSVYPVHKRPLVVRTNVDYEFTTITVDQVTA
ADGNYEVLFLGTDRGTVQKVIVLPSDDLQTEELVLEEVEVFKVPTSITTMKISSKRQQLYVASAVGVTHMALHRCDVYG
EACADCCLARDPYCAWDGKSCSRYSASQKRRSRRQDVKYGNPIRQCRGYNSNINKNTLETVQYGVEGSSTFLECQA
RSPHAAIKWHLHRDNSDRRREVRSDGRVLKTDQGLLLRSLQSSDSGIYICTATEKNFKHTLVKLQLLVLTNQAVNNILVD
TGRPVVSPLQSSAWTPSAGQYKDLLTILSQPEMGLINQYCQDYWQYGDPLTGVIKAKDLKELKEQKKPRNRRHHGDE
EET*

Semaphorin3Fb mutant protein sequence
MLLDSLWPVQVLLVLSVLTLVLGNEPQNAPRVFLSFKDLRSTGTAHYFSFLLNTSDYRILRMDEDHDRMYVGSKDYILSL
DLHDINREPLIIHWPVSPQRKTECVLSGKDTNGECGNFIRLIEPWNRTHLYICGTGAYNPVCTYVNRGRKPMTAMHLQM
PQTRGRASRAAEPETVFDETGLQEYVFHLEPGKEDSGKGKCPYDPKLNSVSALINGELYAGVYIDFMGTDSAIFRTLG
KNAVMRTDQYNSRWLNDPSFVHVHLIPDTVRKTMTNSTFSFVKSLLKWARARNHNPASAASA*TMTVVTAAWSTSGA
PS*KPVSSAQSQELTGLKRTSMSSEMCTYSPRRTLKTQSSTGSSLCPALYSRARRSASTPWRTFGWCSMDLLPTKKV
PITSGWPTLGKSPTPVREHVLVGLSPRT*SPLRTTQTRSSTSCGIIPPCITPCIQCTSAPWWSGLTWTMSSQPSLWIRSQ
RPTETMRCSSWEQTEEQCRR*SSYPVMTCRQRSLCWRKWRSLRSLLLSQQ*RFLQNGNNCMWPLRWE*RIWLCTAV
MSTARPVLTAVWPGTPIVHGTANPALDTQPRRRDAAVDKT*NTGTPYASAEDTIPTLIRTHWRQCSMGWRAAAPFWSA
RPDHHTLPSSGTFTETTATGGEKSAVMAVC*RRIKVCFCALSSPQTPASTSARPQRRTSSTRWSNCSCSS*PIRPSTTF
WWTRDVRSSLRSSPAPGPPVPGNTKTC*PSSASPRWAS*TSTARTIGSMETL*PGSSRPKTSKSSRSRRNHEIADITGT
RRRHE

C

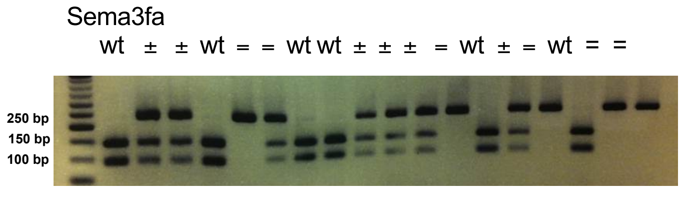

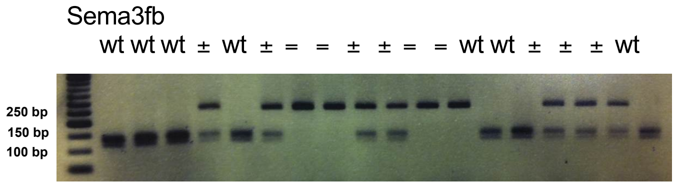

Supplemental Figure 4. Validation of *sema3fa* and *sema3fb* mutant zebrafish lines generated by TALEN genome editing. (A, B) The F1 offspring generated following injection of *sema3fa* and *sema3fb* TALEN constructs into 1-cell stage zebrafish mpx:GFP embryos, were sequenced and (A) Sema3fa and (B) Sema3fb aligned to wild-type, with stop codons represented by *. (C) F1 progeny were in-crossed for inflammation phenotyping, with DNA from each embryo digested for mutation analysis. Representative gel shows examples of wild-type (cleaved, wt), heterozygous (partial cleavage, ±) and homozygous null (uncleaved, =) alleles for individual F1 progeny.

Supplemental Figure 5.

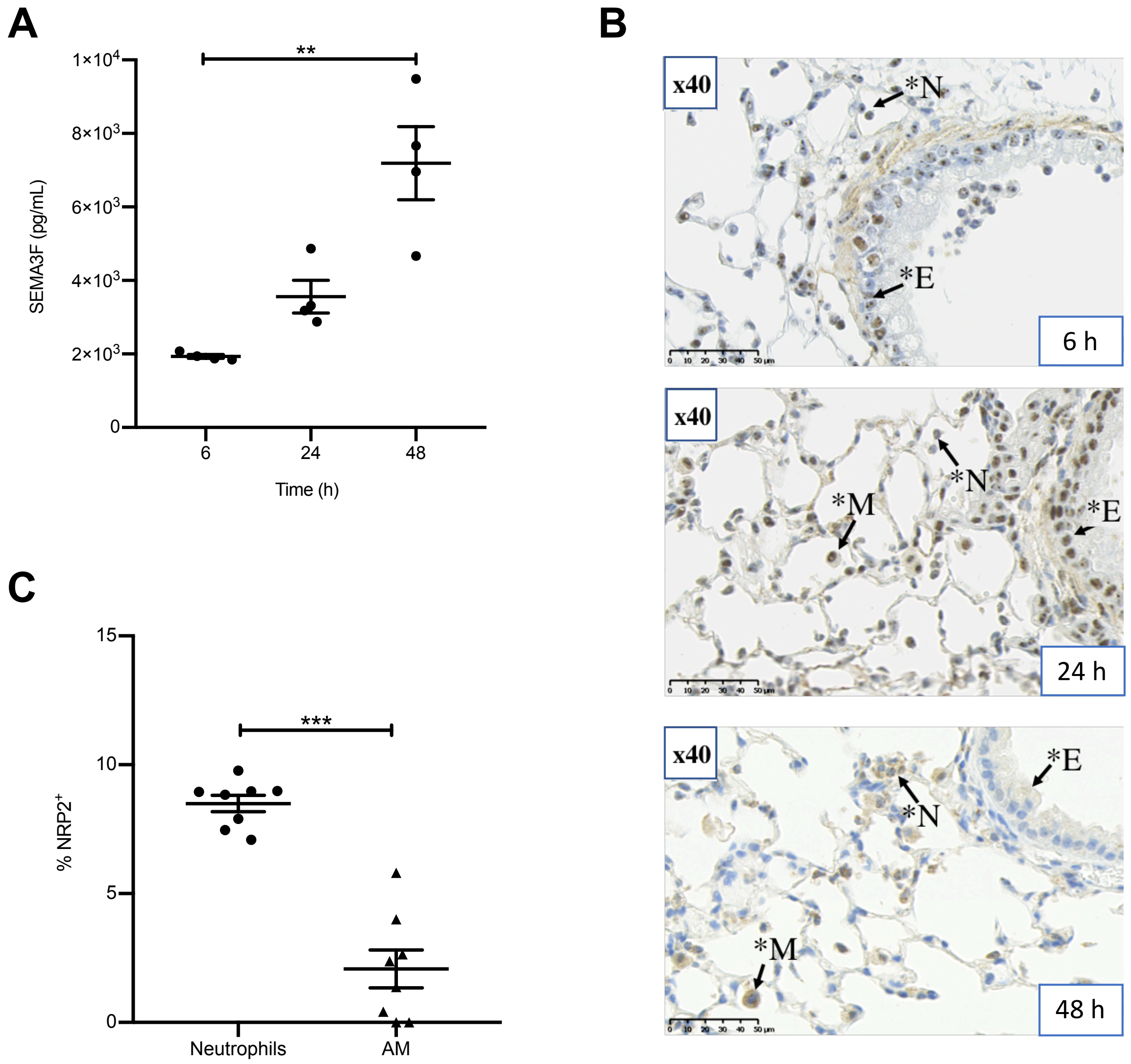

Supplemental Figure 5. Expression of SEMA3F and its co-receptor NRP2 in the airways of a murine model of acute lung injury. An acute lung injury was induced by nebulized LPS, with mice sacrificed and bronchoalveolar lavage (BAL) performed and lung tissue harvested. (A) SEMA3F levels in BAL samples taken at 6, 24 and 48 h following LPS challenge was assessed by ELISA. Data shown are mean ± SEM, n=4. (B) Lung tissue obtained at 6 (top panel), 24 (center panel), and 48 h (bottom panel) following LPS challenge and stained for SEMA3F expression in neutrophils (black arrows *N), epithelium (black arrows *E) and macrophages (black arrows *M). (C) The percentage of airspace neutrophils and alveolar macrophages expressing NRP2 in lung tissue digests was determined by flow cytometry 24 h

post-LPS challenge. Data shown are mean ± SEM, n=8 performed as 2 independent experiments. Statistical analysis: One-way ANOVA and Bonferroni's post hoc test (A) and Kruskal-Wallis and Dunn's post-hoc test (C) were performed; **$P<0.05$, ***$P<0.01$.

Supplemental Figure 6.

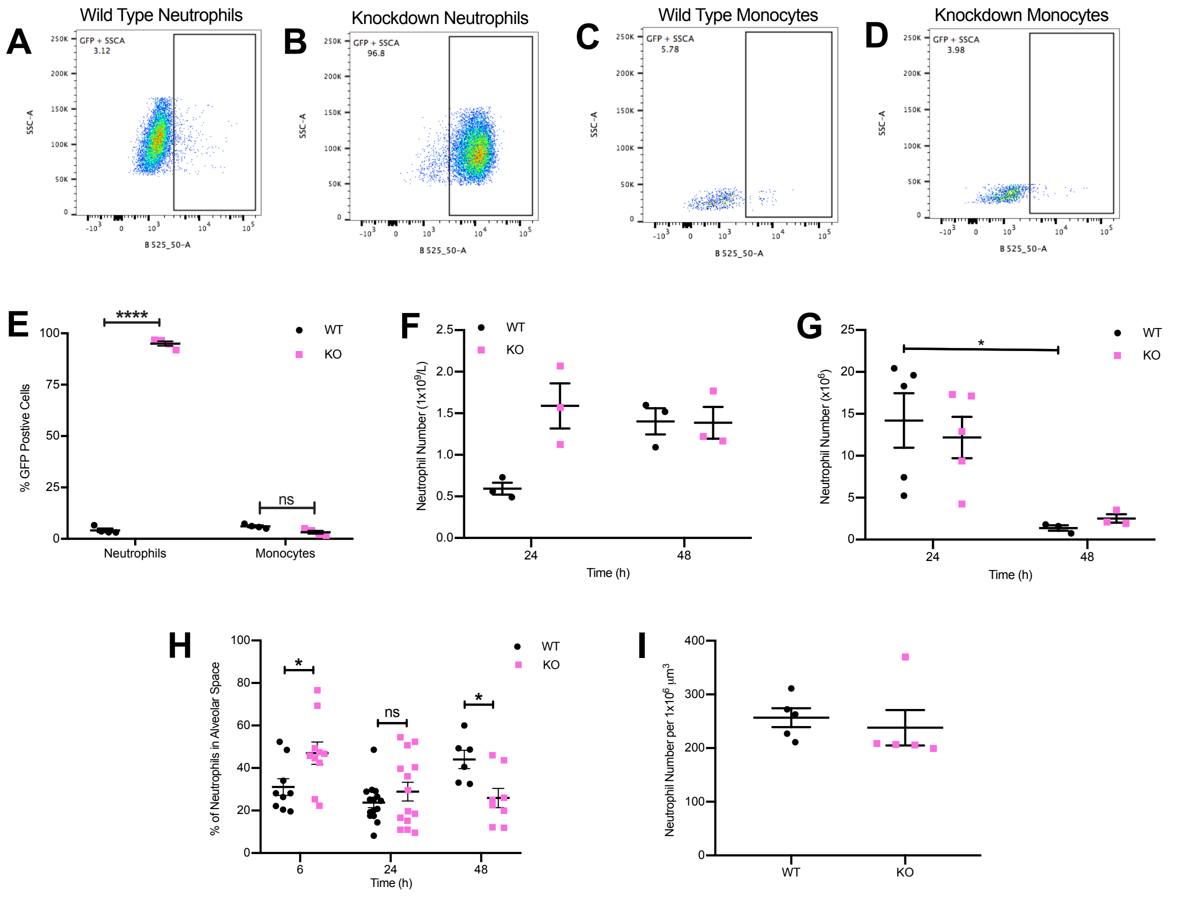

Supplemental Figure 6. Verification of neutrophil knockdown of *Sema3f* and the recruitment and clearance of neutrophils from the lungs in a murine acute lung injury model. *Sema3f*$^{flox/flox}$*Mrp8Cre*$^{-/-}$ (WT) and *Sema3f*$^{flox/flox}$*Mrp8Cre*$^{+/-}$ (KO) mice underwent LPS induced lung injury and were sacrificed at 24 h and bronchioalveolar lavage (BAL) performed. Following identification of the neutrophils and monocyte cell populations in the BAL, the geometric fluorescent intensity of the Green Fluorescent Protein (GFP) signal was determined by flow cytometry. In graphs A-D side scatter intensity is plotted against fluorescent intensity in the GFP channel (B525_50-A). Graphs A and B represent the wild-type and knockdown neutrophil populations respectively; C and D show matching graphs for the monocyte population. The percentage of GFP positive neutrophils and monocytes is shown in graph E, the GFP reporter protein is only significantly expressed in *Sema3f* KO neutrophils. Data shown are mean ± SEM, n=4 performed as an experiment. (F-H) *Sema3f* WT and KO mice were

challenged with nebulised LPS, sacrificed at 6, 24 and 48 h following LPS challenge and blood and lung tissues were harvested with lung digest for Ly6G staining (neutrophil number). Data shown n=3-14. (I) Quantification of neutrophils in stained lung sections harvested from *Sema3f* WT and KO mice at 6 h following LPS challenge. Statistical analysis: Two-way ANOVA and Sidak's post hoc test (E-H) and unpaired *t*-test (I) were performed; *P<0.05, ****P<0.0001.

Supplemental Figure 7.

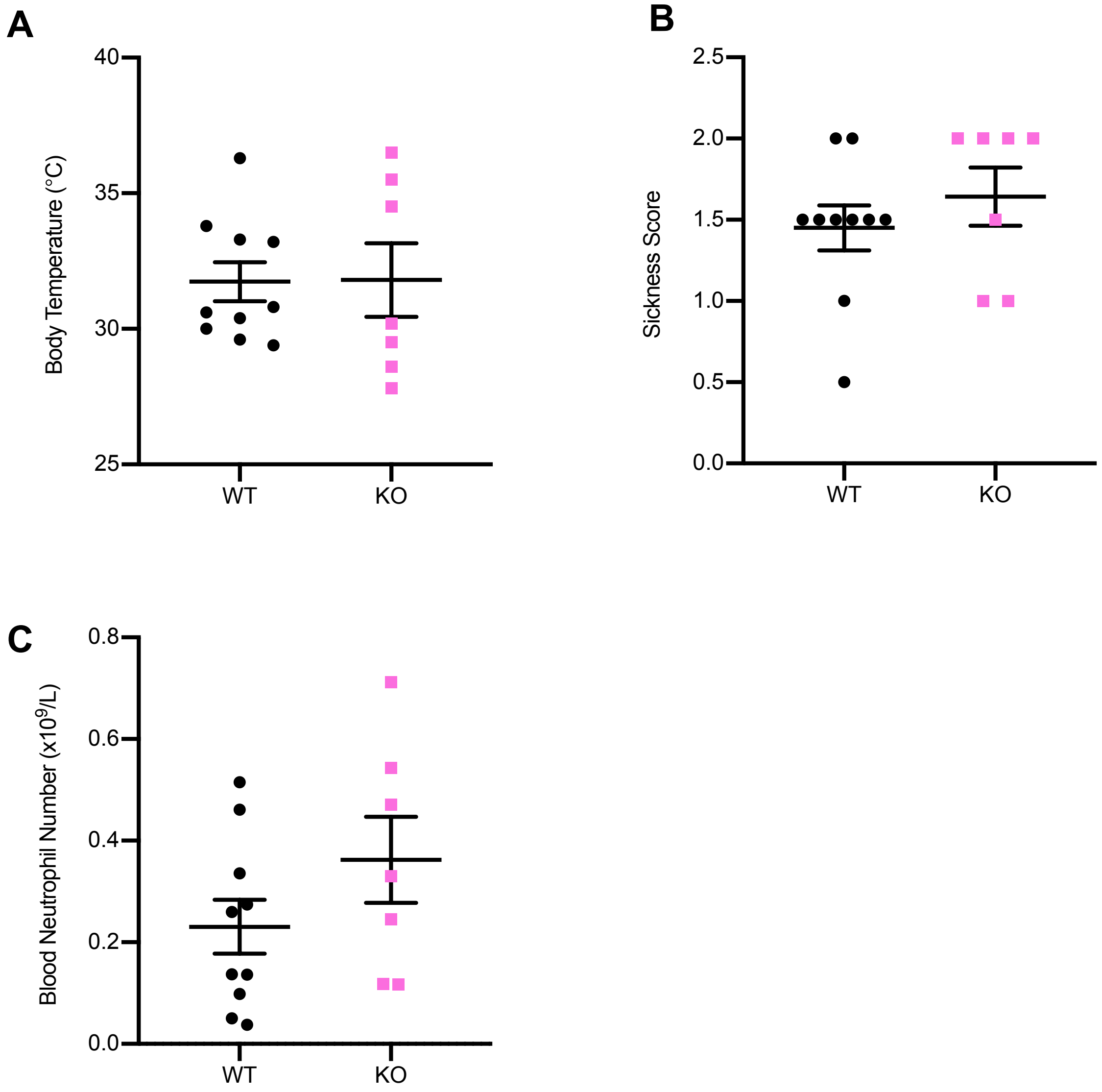

Supplemental Figure 7. Neutrophil-specific knock-out of *Sema3f* does not affect mouse well-being or neutrophil blood counts following *Streptococcus pneumoniae* infection. *Sema3f*^{floxflox}*Mrp8Cre*$^{+/-}$ KO and *Cre*$^{-/-}$ WT mice were challenged with intratracheal instillation of 10^7 *S. pneumoniae* D39 type 2 strain, and body temperature (A), sickness scores (B) and blood neutrophil counts (C) obtained 14 h post instillation. Data shown as mean ± SEM, n=7-10 performed as 2 individual experiments. Statistical analysis: Mann-Whitney (A, B) and unpaired *t*-tests (C) were performed.

Supplemental Figure 8.

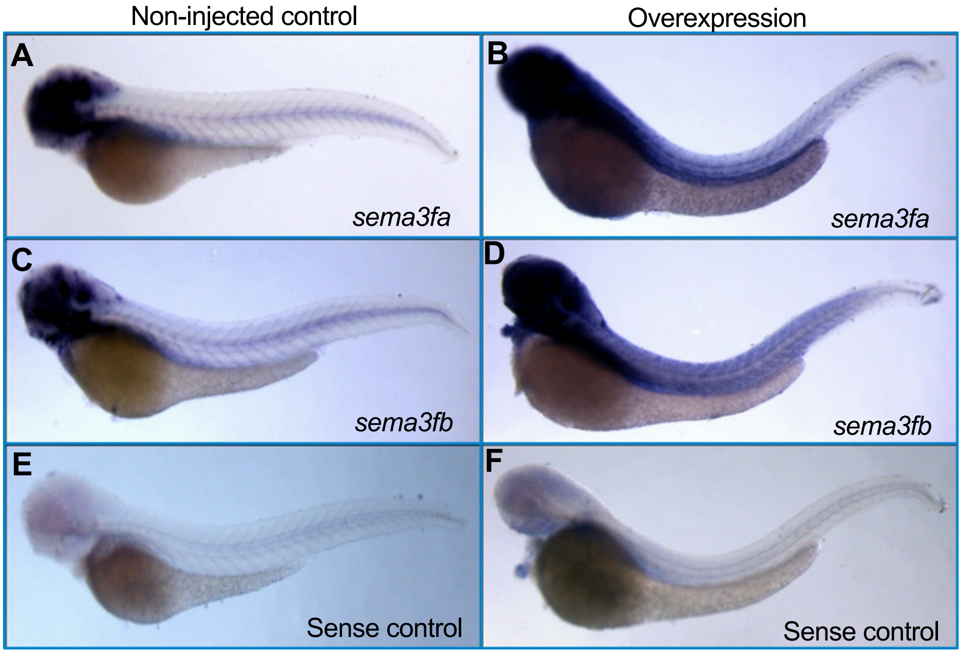

Supplemental Figure 8. Verification of *sema3fa* and *sema3fb* overexpression. *sema3fa* or *sema3fb* RNA (50 ng/μL) was injected into 1-cell stage zebrafish mpx:GFP embryos, 3 dpf embryos fixed in 4% paraformaldehyde, stained with (A-B) *sema3fa* antisense (C-D) *sema3fb* antisense and (E-F) negative control sense probes, with non-injected control embryos processed in parallel (A, C, E).

Supplemental Figure 9.

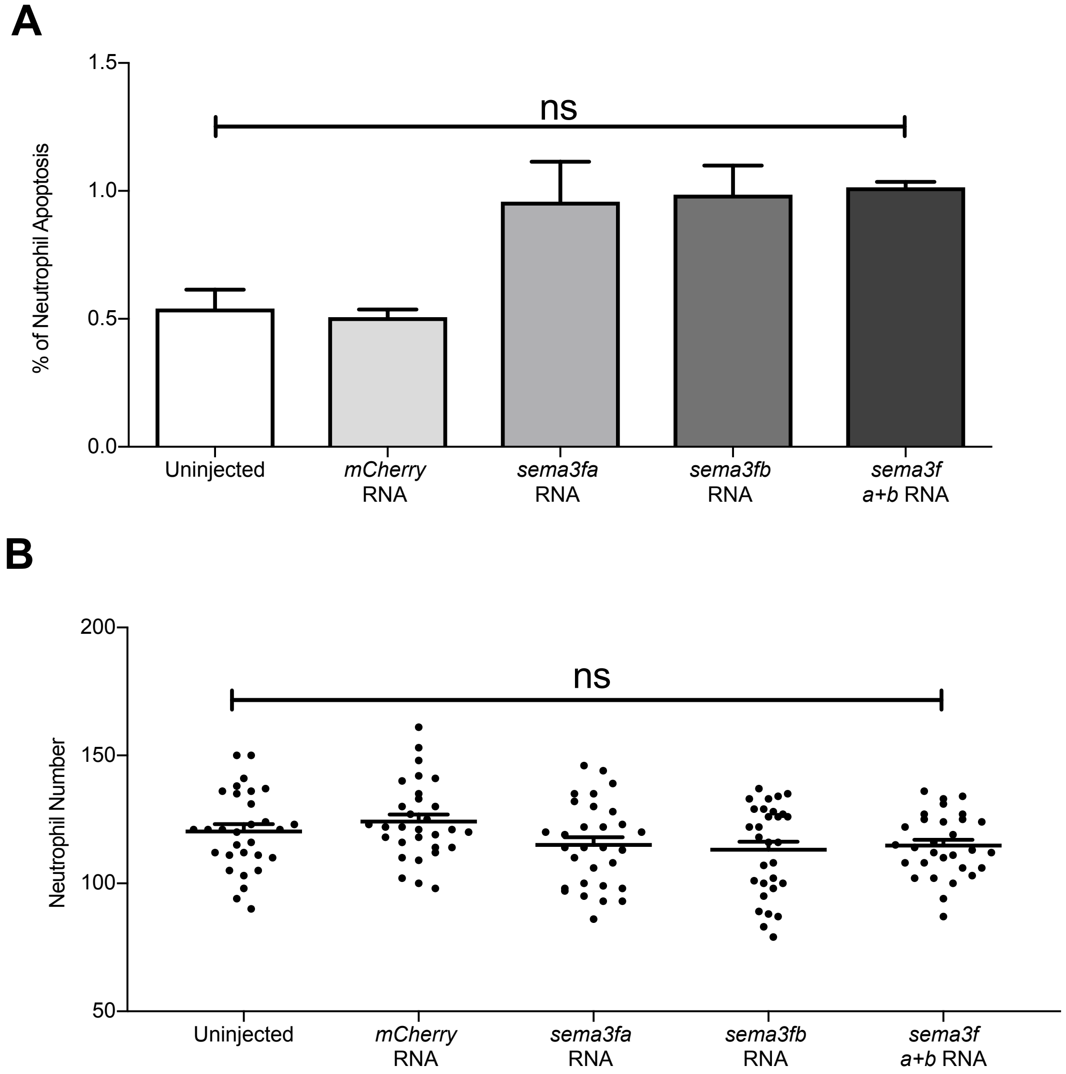

Supplemental Figure 9. Preserved apoptosis responses and total neutrophil counts with *sema3fa* and *sema3fb* mRNA microinjection. *sema3fa*, *sema3fb* or *mCherry* control RNA (50 ng/µl) was injected into 1-cell stage zebrafish mpx:GFP embryos. (A) Tailfin transection was performed at 2 dpf, and at 24 hpi neutrophils stained for terminal deoxynucleotidyltransferase - mediated dUTP nick end labeling and Tyramide signal amplification process (TSA) for endogenous peroxidase activity. Data shown are mean ± SEM, n=180 performed as 3 independent experiments. (B) Whole body neutrophil counts were performed at 3 dpf. Data shown are mean ± SEM, n=30 performed as 3 independent experiments. Statistical analysis: One-way ANOVA and Bonferroni's post hoc test were performed.

Supplemental Figure 10.

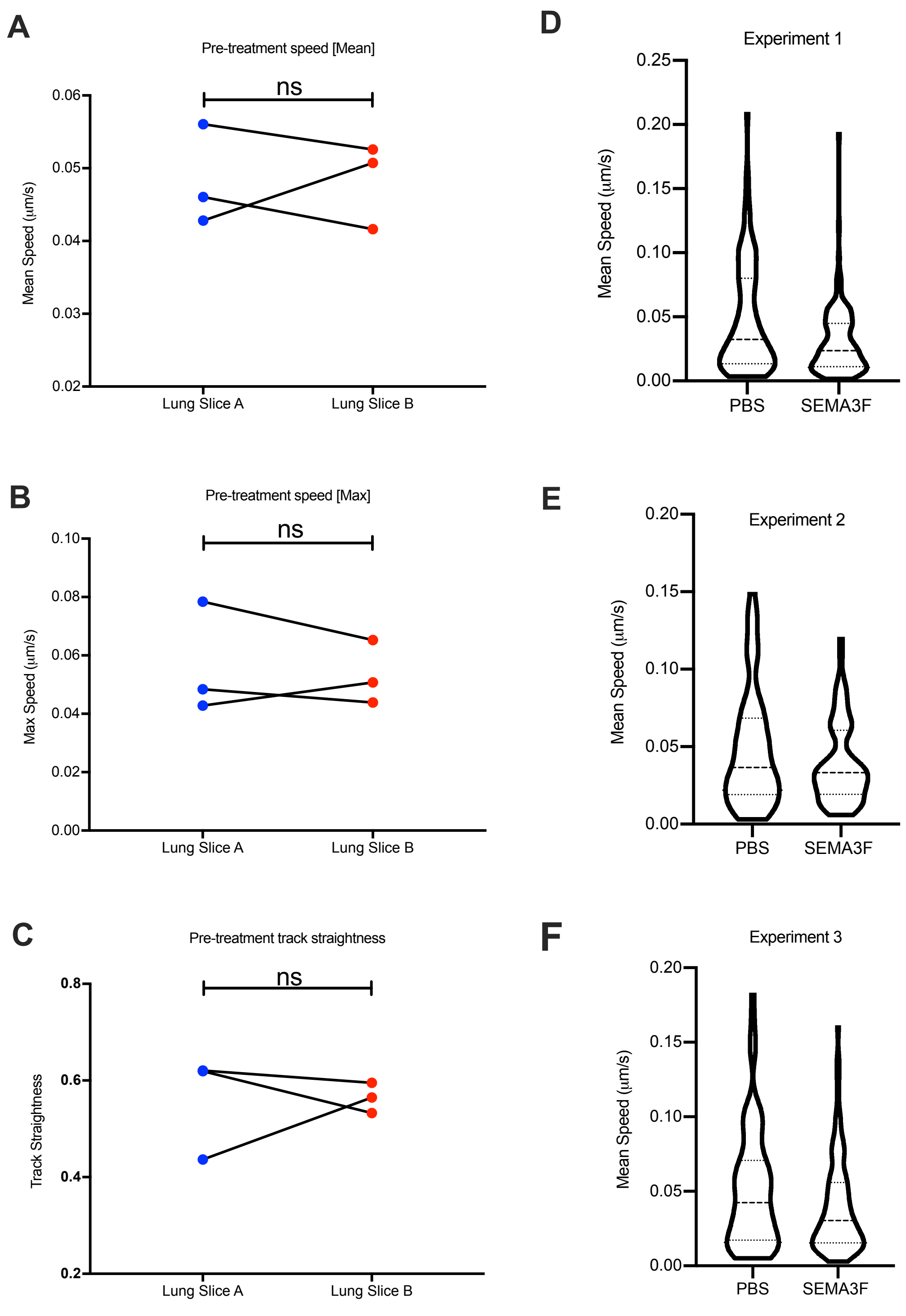

Supplemental Figure 10. Baseline speed and track straightness data for real-time imaging of precision cut lung slices and distribution of speed data for each pair of lung slices. Naïve Catchup$^{IVM\text{-}RED;Lifeact\text{-}GFP}$ mice were sacrificed, lungs instilled with agarose gel, precision sliced and imaged by confocal microscopy for a total of 90 min. After 30 min either SEMA3F

or vehicle control were added to the culture media. For the first 30 min prior to treatment neutrophil mean speed (A), maximum speed (B) and track straightness [directionality] (C) were measured and analysed using Imaris software (Imaris V 9.1). Data shown are n=3 performed as three individual experiments. Violin plots (D-F) show the distribution of mean speed data for each lung slice following treatment with either PBS or SEMA3F 100 nM. Statistical analysis: Paired *t*-tests; *$P<0.05$.

Supplemental Figure 11.

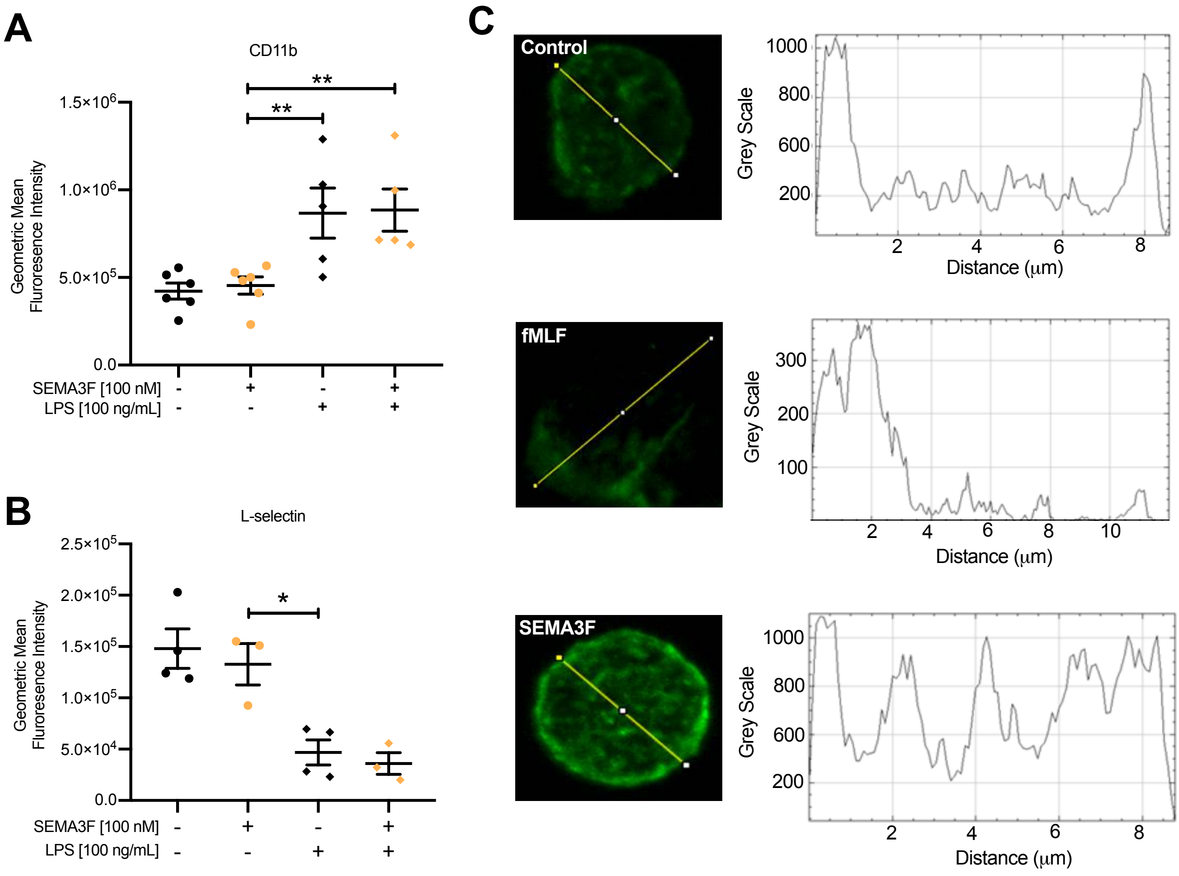

Supplemental Figure 11. Neutrophil treatment with exogenous SEMA3F does not regulate adhesion molecules, but promotes F-actin disassembly. Isolated peripheral blood neutrophils from healthy volunteers were incubated with recombinant SEMA3F [100 nM] and/or LPS [100 ng/mL], stained with PE-anti-CD11b (A) or PE-anti-L-selectin (B) and geometric mean fluorescence determined by flow cytometry. Data shown are mean ± SEM, n=4. (C) Human neutrophils were cultured with PBS, SEMA3F [100 nM] for 60 min or fMLF for 15 min, fixed and stained with Phalloidin and imaged using a laser scanning confocal microscope with 100X objective. Each representative image and histogram shows Phalloidin staining (F-actin) and its distribution in the cell. Statistical analysis: Two-way ANOVA and Sidak's post hoc test were performed; *P<0.05, **P<0.01.

Supplemental Video 1.

Supplemental Video 1. A video demonstrating the systematic approach to analysis of neutrophil position in fixed precision cut lung slices (PCLS) and tracking in live cultured PCLS using IMARIS v9.1 Software. Following fixation, the lung slice was stained with CD31 (green) lung endothelium, S100A9 (red) [neutrophils] and DAPI (blue) [cell nuclei] and imaged as described in the main text. These images were stitched together, an example of an entire lung slice is shown. The lung parenchyma [lung endothelium] is rendered in 3D, seen in grey, a threshold for this was manually adjusted. The neutrophils are identified in red, using the "Spot" function within Imaris. A manual threshold was used to ensure only neutrophils were identified. A mask was created for two neutrophil groups based on their relationship to the rendered 3D lung endothelium. For the internal cell mask all voxels out with the 3D structure were given the value of 0, for the external cell mask all voxels within the 3D lattice were ascribed a value of 0. Using these masks spots were rendered: the purple spots represent the internal cells and the yellow spots the external cells. As an example, in the video only the yellow (external cells) are visible outside the grey lattice. The points where internal and external cells overlapped were termed colocalization and denote the peri-vascular position.

Supplemental Video 2.

Supplemental Video 2. A video demonstrating the systematic approach to analysis of neutrophil tracking in live cultured PCLS using IMARIS v9.1 Software. In the live lung slice culture system neutrophils were identified by their transgenic fluorescence [Catchup$^{\text{IVM-RED;Lifeact-GFP}}$ mouse] and are shown in red in Supplemental Video 2. For each frame neutrophils were identified using the "Spot" function and tacked over time using an autoregression movement pattern. The frames were manually inspected, underwent a manual threshold and any aberrant tracking corrected. Shown in the video are the neutrophil signals and tracking using spots with colored comet tails which depict the historic journey of the cell over time.
